# A Direct Arylation Approach for Helicene Analogues Composed of Unidirectional Amide Linkages

**DOI:** 10.1021/acsomega.6c06995

**Published:** 2026-08-20

**Authors:** Akihiro Yokoyama, Hirotaka Shimoda, Shoko Kikkawa, Isao Azumaya, Taro Tsubomura, Kosuke Oki

**Affiliations:** † Faculty of Science and Technology, 13038 Seikei University, 3-3-1 Kichijoji-kitamachi, Musashino, Tokyo 180-8633, Japan; ‡ Faculty of Pharmaceutical Sciences, Toho University, 2-2-1 Miyama, Funabashi, Chiba 274-8510, Japan

## Abstract

Amide-bond-containing [5]­helicene and [7]­helicene analogues were synthesized via palladium-mediated 2- and 3-fold direct intramolecular arylations of halogenated linear diamides and triamides, respectively. The orientation of the amide bonds in these helicene analogues was designed to align with the winding direction of helicene. The introduction of a methyl group at the 3-position of the terminal aniline unit of the linear triamide precursor is crucial to the synthesis of the [7]­helicene analogue. The molecular structures of the [5]­helicene and [7]­helicene analogues in the crystalline state were determined unequivocally by single-crystal X-ray diffraction analysis. The addition of trifluoroacetic acid to a solution of the [7]­helicene analogue in dichloromethane increased the fluorescence intensity, whereas the absorption intensity remained unchanged. The enantiomers of the [7]­helicene analogue were separated by high-performance liquid chromatography over a chiral stationary phase. The enantiomers exhibited circularly polarized luminescence with relatively large luminescence dissymmetry factors.

## Graphical abstract

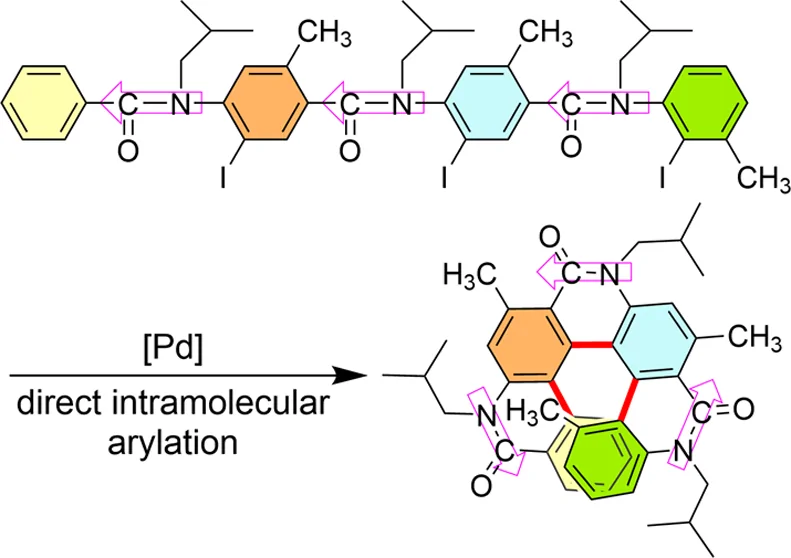

## 1 Introduction

Carbohelicenes and heteroatom-containing helicene-like molecules have attracted increased attention due to their unique properties arising from their twisted structures.^1–5^ To synthesize the helicene structure, a variety of methods have been developed including photocyclization,^6–9^ Friedel–Crafts acylation,^10^ Diels–Alder reaction,^11–16^ radical cyclization,^17^ alkyne benzannulation,^18,19^ NH/CH oxidative coupling reactions,^20^ [2 + 2 + 2] cycloaddition,^21–24^ amidation,^25,26^ hydroarylation,^27,28^ and direct arylation.^29^ Of these reactions, the [2 + 2 + 2] cycloaddition has been used for the enantioselective synthesis of helicene-like molecules.^30–32^

Aromatic amides are frequently used building blocks in various molecular architectures. The helically folded conformation of several aromatic oligoamides in which pairs of benzene rings are connected by single N–H or N-alkylated amide linkage has been reported.^33^ Amide linkages have also been used to construct helicene frameworks that adopt configurationally stable helical structures, and several helicene-like compounds containing one^16,25–27,34,35^ or two^14,28^ amide bonds have been synthesized (Figure 1). [6]­Helicene analogue **A** ^27^ and [10]­helicene analogue **B** ^28^ are representative examples that can be synthesized enantioselectively via the Au-catalyzed sequential intramolecular hydroarylation of alkynes. Compound **C**, which bears an N–H type amide bond, was obtained from the photocyclization of a boron hydroxamate complex using a continuous flow strategy.^16^ Photoreactions have also been used for the synthesis of a series of [*n*]­helicene analogues **D** (*n* = 0–3)^34^ and **E**.^14^ An additive- and ligand-free Pd-catalyzed domino coupling reaction was used to generate [5]­helicene analogue **D** (*n* = 1),^35^ while [7]­helicene analogues **F** and **G** were synthesized via an intramolecular amidation.^25,26^ For some of these compounds, the solid-state structure has been confirmed by single-crystal X-ray diffraction analysis, and the chiroptical properties have been studied using circular dichroism (CD) spectroscopy. Recently, circularly polarized luminescence (CPL) spectra have been used to explore the chiroptical properties of helicenes, albeit that the CPL spectra of amide-containing helicenes have not yet been reported. Furthermore, the synthesis of helicene-like compounds with three amide bonds aligned with the winding direction of the helicene remains a challenging task.

**Figure 1:**
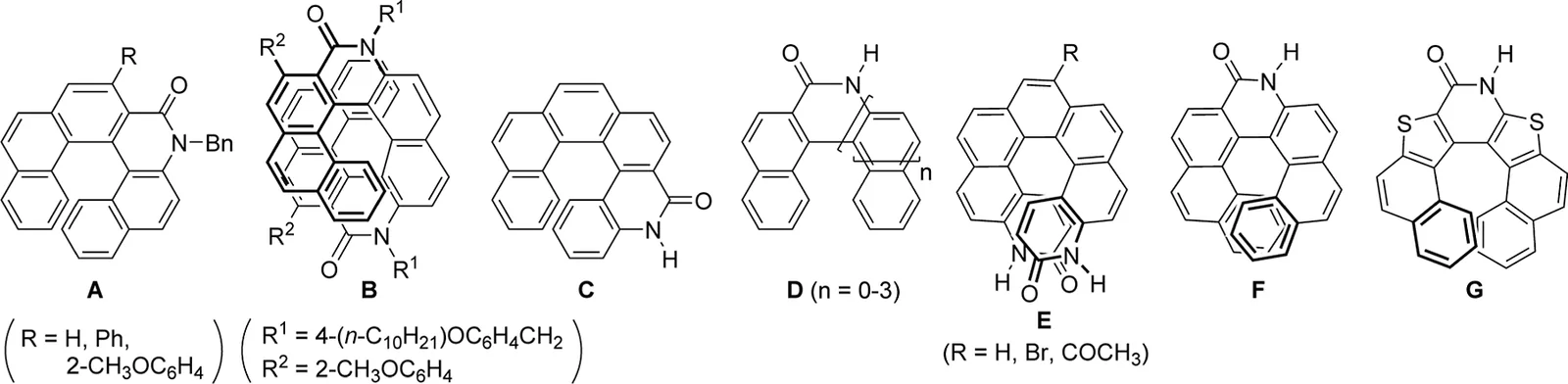
Hitherto reported amide-containing helicenes.

In the course of our studies on the synthesis of amide-containing polycyclic aromatic molecules via a fold-in approach, we have synthesized coronene^36,37^ and ovalene^38^ analogues through the Pd-mediated direct intramolecular arylation of N-alkylated cyclic aromatic oligoamides. We envisioned that helicene-like structures, in which the orientation of the amide bonds is consistent with the winding direction of the helicene, could potentially be constructed by applying a direct intramolecular arylation reaction to linear aromatic oligoamides composed of 4-(alkylamino)­benzoic acids. Here, we report the synthesis and properties of [5]­helicene and [7]­helicene analogues **1** and **2** formed by direct intramolecular double and triple arylation of linear diamide **3** and triamide **4**, respectively (Scheme 1).

**Scheme 1:**
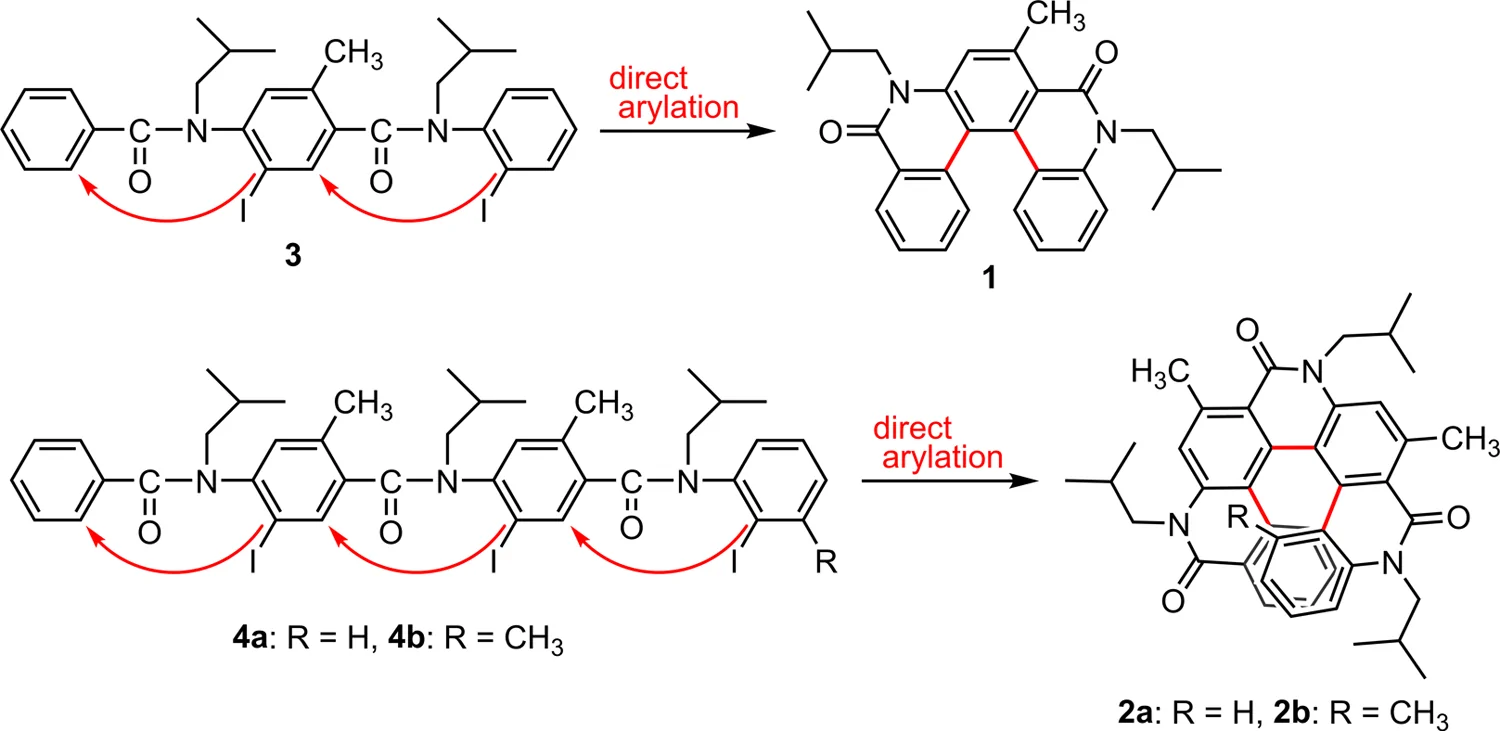
Synthesis of [5]­Helicene and [7]­Helicene Analogues **1** and **2**

## 2 Results and Discussion

### 2.1 Synthesis

To optimize the reaction conditions of the direct intramolecular arylation, we first studied the synthesis of **1**. The linear precursor to **1**, i.e., **3**, was synthesized as shown in Scheme 2. The reaction between **5** ^39^ and isobutyraldehyde in the presence of NaBH­(OAc)_3_ ^40^ afforded *N*-isobutyl product **6** in 96% yield, and subsequent reaction with benzoyl chloride (BzCl) furnished amide **7** in 96% yield. After hydrolysis with KOH (96% yield), carboxylic acid **8** was treated with SOCl_2_, followed by treatment with 2-iodo-*N*-isobutylaniline (**9**) to give diamide precursor **3** in 91% yield. The ^1^H NMR spectrum of **3** in CDCl_3_ at room temperature exhibited highly complex signals owing to slow rotation around the amide–aryl bonds together with slow *cis*/*trans* conformational interconversion of the amide groups on the NMR time scale. Upon heating to 150 °C in DMSO-*d*_6_, the signals became sufficiently averaged to afford a broadened but analyzable spectrum.

**Scheme 2:**
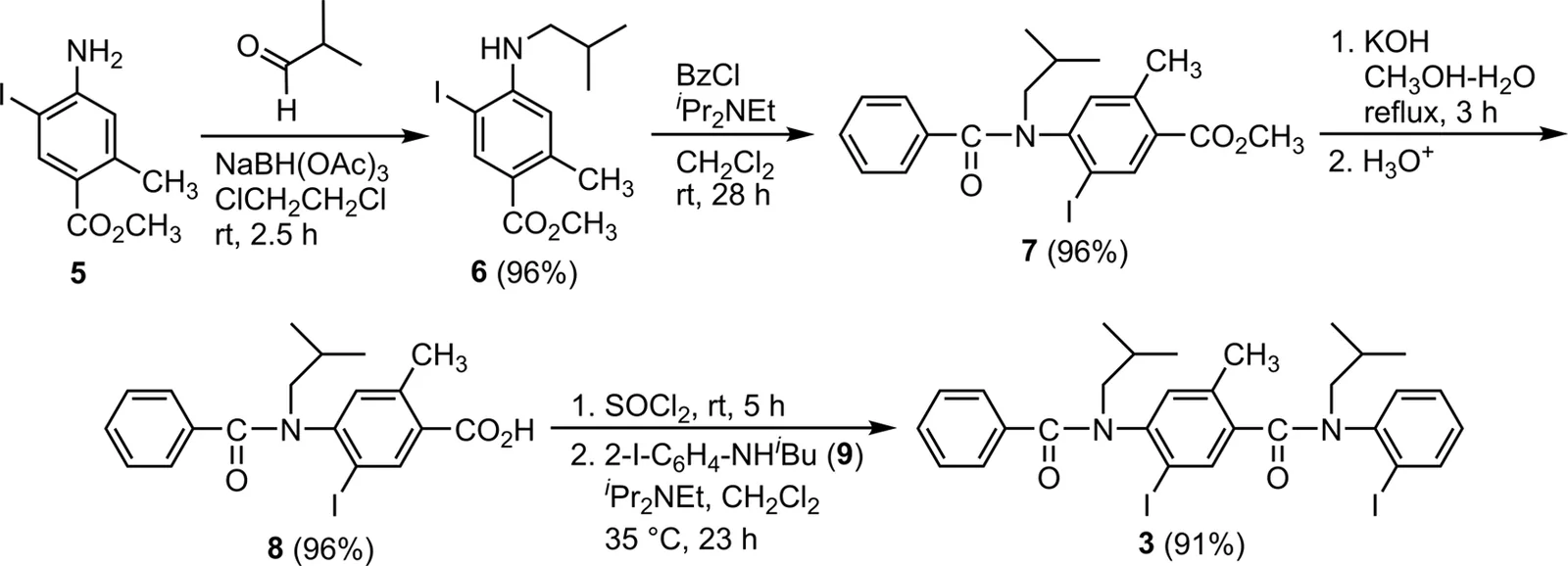
Synthesis of Diamide **3**

Subsequently, we employed the reaction conditions reported by Harayama and co-workers^41^ to investigate the direct intramolecular arylation of **3** using a combination of Pd­(OAc)_2_ and PPh_3_ in the presence of K_2_CO_3_ in DMF (Scheme 3). The results of the optimization of reaction conditions are summarized in Table 1. The reaction of **3** with 0.2 equiv of Pd­(OAc)_2_, 0.4 equiv of PPh_3_, and 4 equiv of K_2_CO_3_ at reflux temperature for 2 h afforded **1** in 55% yield, together with palladium complex **10** in 14% yield (Table 1, entry 1). The structures of these products were supported by the spectroscopic data and finally confirmed by X-ray crystallographic analyses (Figure 2A,B).^42^ Lowering the reaction temperature to 100 °C decreased the yield of **1** to 30% (Table 1, entry 2), while the use of twice the amount of Pd­(OAc)_2_ (0.4 equiv) and PPh_3_ (0.8 equiv) improved the yield of **1** to 60% (Table 1, entry 3). Finally, the reaction at 80 °C for 44 h afforded **1** in 73% yield (Table 1, entry 4).

**Scheme 3:**
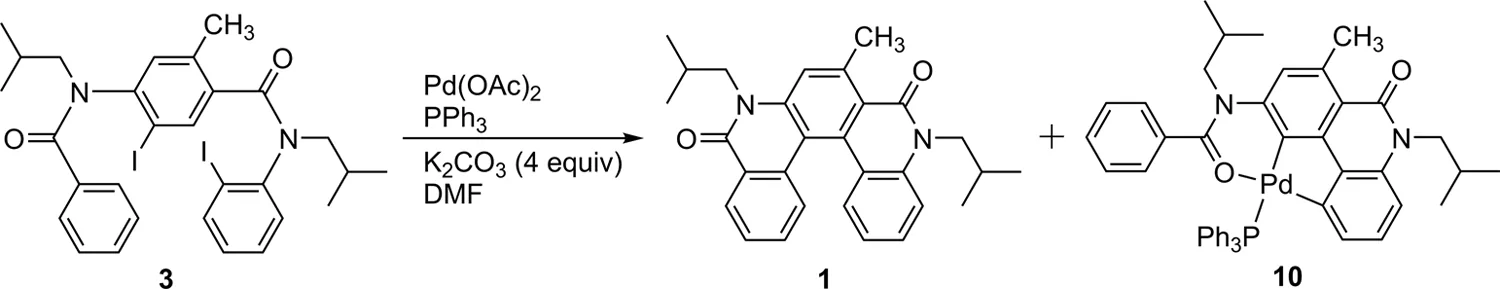
Direct Intramolecular Arylation of **3**

**Table 1:** Optimization of the Direct Intramolecular Arylation of **3** ^a^

entry	Pd(OAc)_2_ (equiv)	PPh_3_ (equiv)	temp	time (h)	yield of **1** (%)	yield of **10** (%)
1	0.2	0.4	reflux	2	55	14
2	0.2	0.4	100 °C	21	30	6
3	0.4	0.8	100 °C	17	60	11
4	0.4	0.8	80 °C	44	73	0

a Reaction conditions: **3** (0.15 mmol), Pd­(OAc)_2_, PPh_3_, and K_2_CO_3_ (0.62 mmol, 4 equiv) in DMF (1.6 mL) in a 5 mL brown round-bottom flask under an argon atmosphere.

**Figure 2:**
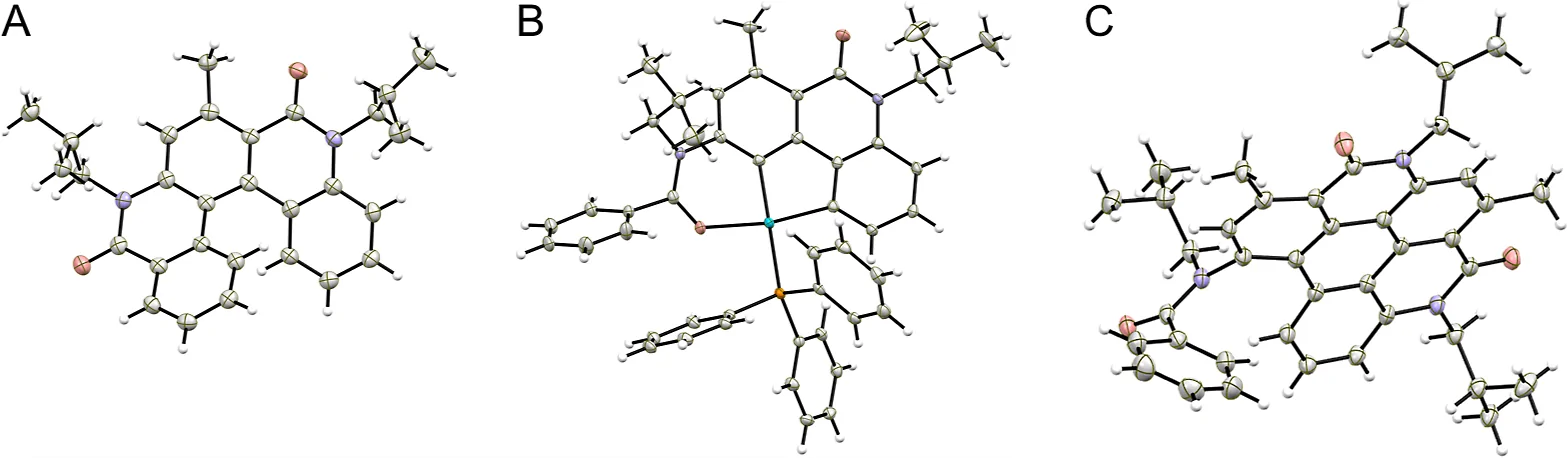
Molecular structures of (A) (*P*)-**1**, (B) **10**, and (C) **13** in the crystalline state with thermal ellipsoids at 50% probability. Solvent molecules are omitted for clarity. Single crystals of **1**, **10**, and **13** suitable for X-ray diffraction analysis were obtained via the slow diffusion of hexane into a dichloromethane solution of racemic **1**, **10**, and **13**, respectively. In the crystal of **1**, the unit cell contained two pairs of *M*- and *P*-helical structures.

We next synthesized linear triamide **4a** as a precursor of [7]­helicene analogue **2a** (Scheme 4). In analogy to the synthesis of **3**, carboxylic acid **8** was converted into the acid chloride through reaction with SOCl_2_, and then treatment with **6** afforded **11** in 91% yield. The hydrolysis of **11** quantitatively furnished carboxylic acid **12**, and **4a** was obtained in a yield of 76% through the condensation of **12** and **9** via an acid chloride intermediate.

**Scheme 4:**
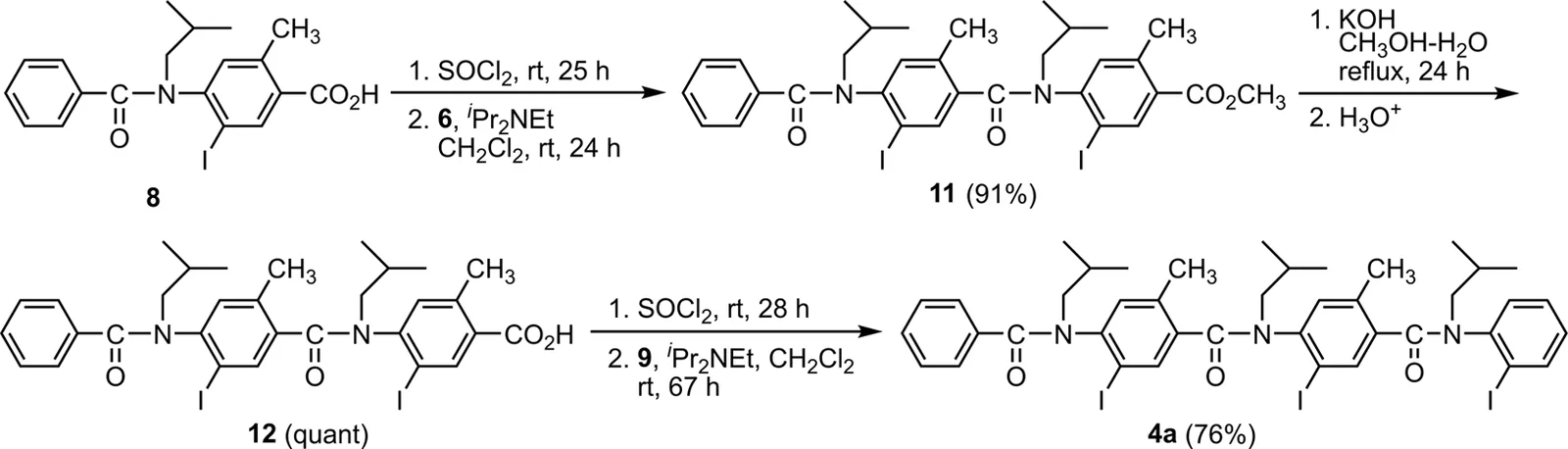
Synthesis of Triamide **4a**

The direct intramolecular arylation of **4a** was conducted using the optimized conditions for the conversion of **3** to **1** (Table 1, entry 4) with 1.5 times the amount of the reagents based on the number of the reaction sites of **4a**. When **4a** was treated with 0.6 equiv of Pd­(OAc)_2_ and 1.2 equiv of PPh_3_ in the presence of 6 equiv of K_2_CO_3_ in DMF at 80 °C for 48 h, [7]­helicene analogue **2a** was not formed, but benzo­[*ghi*]­perylene analogue **13** was obtained in 17% yield (Scheme 5). The structure of **13** was examined by means of ^1^H NMR and ^13^C NMR spectroscopy as well as high-resolution mass spectrometry. Its solid-state structure was confirmed by X-ray crystallography of a single crystal obtained from recrystallization of **13** at room temperature from a dichloromethane solution slowly infused with hexane (Figure 2C). As shown in Scheme 5, the formation of **2a** requires an intramolecular arylation between the A ring and the B ring. The increasing steric repulsion during the formation of **2a** would most likely prevent the C–H moiety on the A ring from reacting. Instead, the C–H group on the D ring could react to give **13**.

**Scheme 5:**
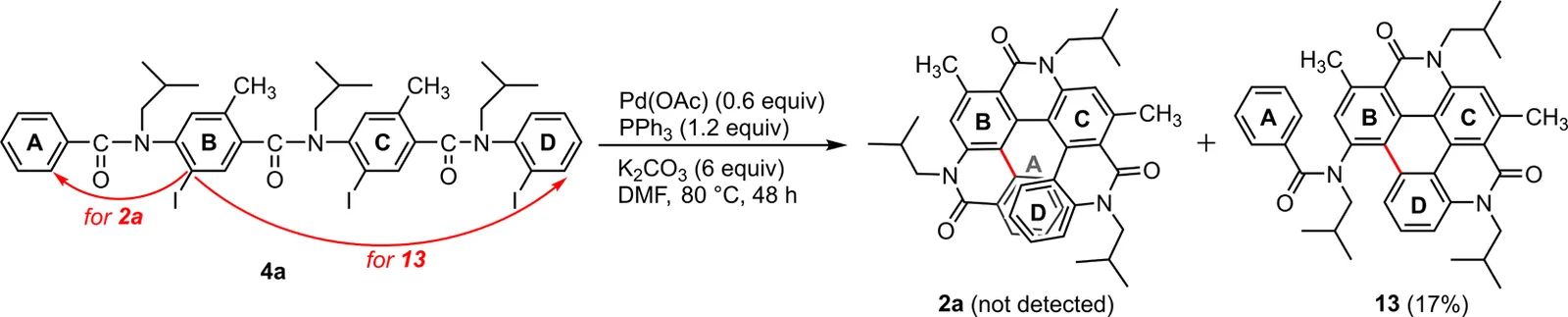
Direct Intramolecular Arylation of **4a**

Harayama’s group proposed that the Pd-mediated direct intramolecular arylation of *N*-(2-iodophenyl)-*N*-methylbenzamide proceeded via a C–H activation mechanism.^41^ Therefore, we expected that replacing the hydrogen atom at the 3-position of the terminal aniline unit in **4a** with a methyl group would suppress the reaction at that position. As shown in Scheme 6, linear triamide **4b** was synthesized via the condensation of **14** and an acid chloride intermediate, prepared by the reaction of **12** and SOCl_2_. We next investigated the intramolecular biarylation of **4b**, and the results are summarized in Table 2. When the reaction of **4b** was carried out under the same conditions as for the reaction of **4a** (0.60 equiv of Pd­(OAc)_2_, 1.2 equiv of PPh_3_, 6.0 equiv of K_2_CO_3_, DMF, 80 °C) with an initial concentration of **4b** of 60 mmol/L, TLC analysis of the crude products indicated the formation of several types of products. Separation by silica gel column chromatography revealed that target [7]­helicene analogue **2b** was obtained in 4% yield, together with byproduct **15** in 57% yield (Table 2, entry 1). In this reaction, the formation of benzo­[*ghi*]­perylene derivatives like **13** was not observed. This indicates that **13** was formed as shown in Scheme 5 and that the introduction of a methyl group suppressed the formation of **13** as expected. The solid-state structures of **2b** and **15** were confirmed by X-ray crystallography (Figure 3).^43^ Compound **15** is likely formed by the reaction between the palladium-bonded carbon atom on the C ring and the methyl group on the D ring of **4b**, probably due to the increase in steric interaction that occurs during the formation of the [7]­helicene skeleton. A decrease of the initial concentration of **4b** to 10 mmol/L (Table 2, entry 2) and 5.0 mmol/L (Table 2, entry 3) increased the yield of **2b** to 14% and 26%, respectively. The use of Ag_2_CO_3_ as a base^44^ instead of K_2_CO_3_ did not improve the yield of **2b** (17%; Table 2, entry 4). We next investigated the effect of the ligands. When P­(*o*-tol)_3_ ^45^ (Table 2, entry 5) or 1,2-bis­(diphenylphosphino)­ethane^46^ (dppe; Table 2, entry 6) were used as ligands, the results were poor. However, the reactions with 1,3-bis­(diphenylphosphino)­propane (dppp) and PBu_3_ as the ligands in combination with Ag_2_CO_3_ as the base^47^ furnished **2b** in 29% yield (Table 2, entry 7). The use of tricyclohexylphosphonium tetrafluoroborate^48^ resulted in a low yield of **2b** (7%; Table 2, entry 8). In each of the reactions of **4b** shown in entries 1–8 of Table 2, the crude products contained many unidentified compounds. Therefore, it appears difficult to further improve the yield of **2b** using known Pd-mediated systems. Compared to the synthesis of the amide-containing helicene analogues **A–G** described in the introduction, our synthetic method allowed us to construct a helicene structure in which the three amide bonds aligned with the winding direction, requiring a comparable or only slightly greater number of steps.

**Scheme 6:**
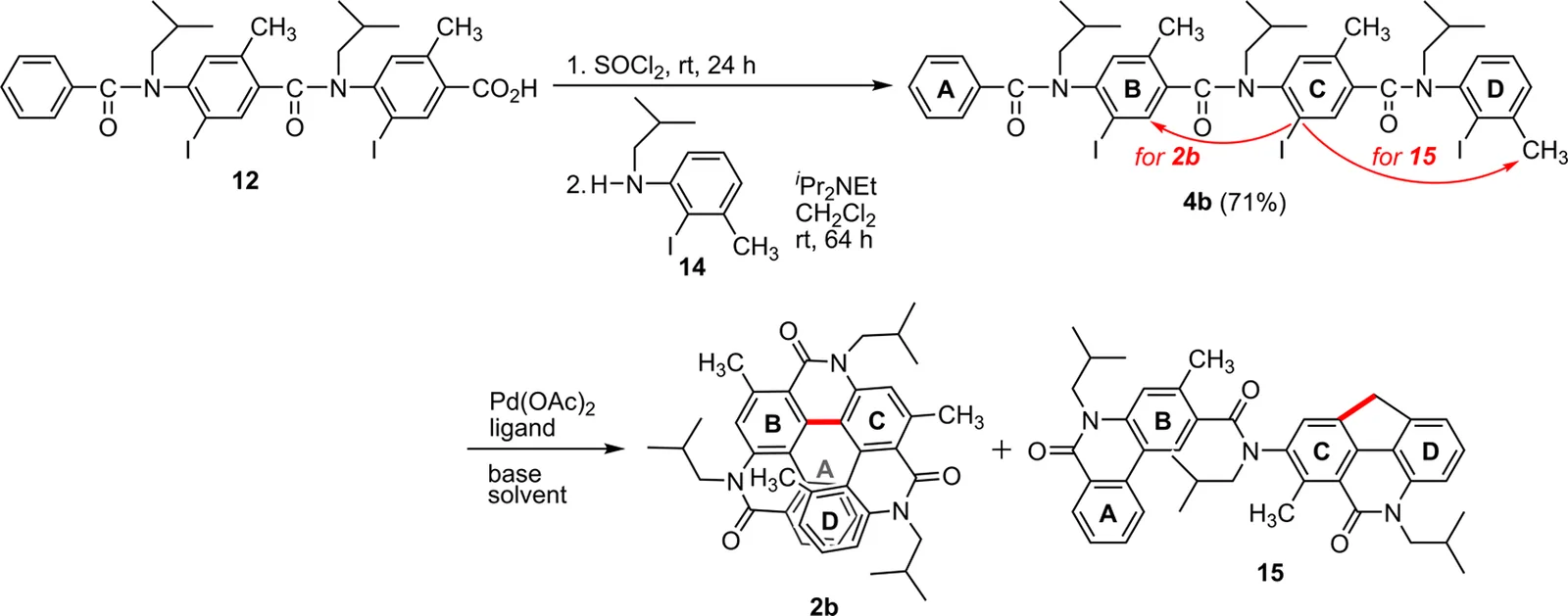
Synthesis and Direct Intramolecular Arylation of Triamide Precursor **4b**

**Table 2:** Optimization of the Direct Intramolecular Arylation of **4b** ^a^

entry	[**4b**]_0_ (mmol/L)	Pd(OAc)_2_ (equiv)	ligand (equiv)	base (equiv)	temp	time (h)	yield of **2b** (%)	yield of **15** (%)
1	60	0.60	PPh_3_ (1.2)	K_2_CO_3_ (6.0)	80 °C	66	4^b^	57
2	10	0.60	PPh_3_ (1.2)	K_2_CO_3_ (6.0)	80 °C	62	14	41
3	5.0	0.59	PPh_3_ (1.2)	K_2_CO_3_ (6.0)	80 °C	62	26	47
4	30	0.67	PPh_3_ (1.2)	Ag_2_CO_3_ (5.9)	reflux	0.25	17^b^	0
5	5.1	0.27	P(*o*-tol)_3_ (0.6)	K_2_CO_3_ (6.0)	reflux	0.5	8^b^	48
6	82	0.16	dppe (0.15)	K_2_CO_3_ (6.0)	reflux	0.5	0	0
7	31	3.0	dppp (3.0), PBu_3_ (3.3)	Ag_2_CO_3_ (5.9)	reflux	0.25	29^b^	22^b^
8^c^	100	0.15	PCy_3_·HBF_4_ (0.31)	Ag_2_CO_3_ (1.5), K_2_CO_3_ (6.0)	130 °C	14	7^b^	0

a Reaction conditions: **4b**, Pd­(OAc)_2_, ligand, and base in DMF in a brown round-bottom flask under an argon atmosphere.

b It contained a small amount of impurities.

c DMAc was used as the solvent.

**Figure 3:**
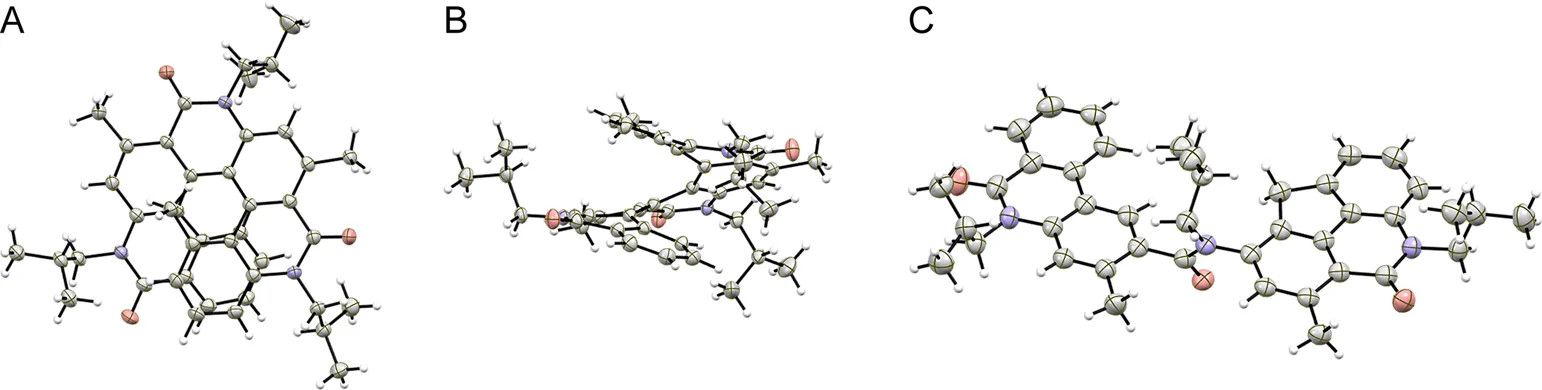
(A) Top view and (B) side view of the molecular structures of (*M*)-**2b**, as well as of (C) **15** with thermal ellipsoids at 50% probability. Solvent molecules are omitted for clarity. In the crystal of **2b**, the unit cell contained two pairs of *M*- and *P*-helical structures.

### 2.2 Structure and Properties

A single crystal of **2b** suitable for X-ray diffraction analysis was obtained from the slow diffusion of hexane into a dichloromethane solution of **2b**. The unit cell contains two pairs of *M*- and *P*-helical structures. The X-ray diffraction analysis revealed that the terminal benzene rings overlap (Figure 3A) and that the molecule has a screw-shaped structure (Figure 3B). The interplanar angle of the terminal benzene rings (17.3°) is smaller than that of carbo[7]­helicene (30.7°–33.8°)^49–51^ but close to that of a hexahydro[7]­helicene,^52^ probably due to the flexible nature of the amide C–N bonds.

The absorption and fluorescence spectra of **2b** were measured in chloroform at room temperature at a concentration of 1.0 × 10^–5^ M (Figure 4A). Absorption maxima were observed at 260 and 298 nm with a shoulder at 403 nm. The fluorescence maximum was observed at 488 nm with a shoulder at 416 nm, and the fluorescence quantum yield (Φ) in chloroform was determined to be 3%, which is similar to that of carbo[7]­helicene.^53^ When the concentration of **2b** was increased from 1.0 × 10^–6^ M to 3.0 × 10^–5^ M, the absorbance intensities increased proportionally (Figure S1A). However, increasing the concentration of **2b** above 1.5 × 10^–5^ resulted in only a slight increase of the fluorescence intensity, probably due to concentration quenching (Figure S1B). The absorption and fluorescence spectra of **2b** were not affected by the solvent, and almost the same spectra were observed in chloroform, dichloromethane, acetonitrile, tetrahydrofuran (THF), and hexane (Figure S2). Inspired by the outstanding work of Otani and Shibata,^20^ we next examined the effect of the addition of trifluoroacetic acid (TFA) on the measured spectra. As shown in Figure 4B,C, addition of 200 equiv of TFA to a solution of **2b** in dichloromethane caused no significant changes in the absorption and fluorescence spectra. When 2000 equiv of TFA was added, the fluorescence intensity and quantum yield (Φ = 12%) increased, whereas the absorption intensity remained essentially unchanged. Similar to when *N*-methyl-1,8-naphthalimide is protonated,^54^ the hydrogen bonding between TFA and the oxygen atoms of the amide bonds in **2b** might decrease the efficiency of the intersystem crossing of **2b**. In the case of *N*-methyl-1,8-naphthalimide^55^ and Si-substituted coumarins,^56^ the suppression of intersystem crossing via hydrogen bonding was supported by theoretical calculations.

**Figure 4:**
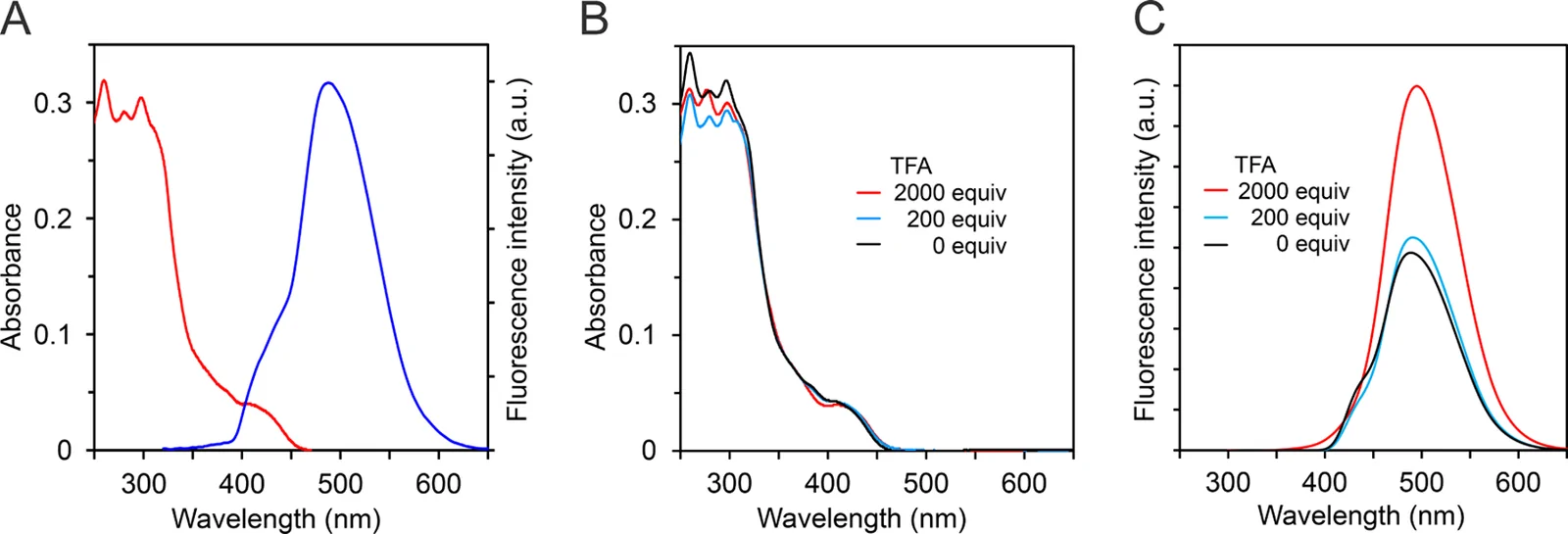
(A) Absorption (red line) and fluorescence (blue line, λ_ex_ = 298 nm) spectra of **2b** in chloroform at room temperature ([**2b**] = 1.0 × 10^–5^ M). (B) Absorption and (C) fluorescence spectra of **2b** in dichloromethane at room temperature in the absence of TFA (black line) and in the presence of 200 equiv of TFA (blue line) or 2000 equiv of TFA (red line) ([**2b**] = 1.0 × 10^–5^ M).

Next, the enantiomers of **2b** were resolved from a racemic mixture using high-performance liquid chromatography (HPLC) over a chiral stationary phase. The specific rotation of enantiopure (+)-**2b** showed a [α] value of +1405 (*c* = 0.10 g/dL, CHCl_3_) which is approximately one-fourth that of carbo[7]­helicene.^57^ The CD and CPL spectra of (+)-**2b** and (−)-**2b** in chloroform are shown in Figure 5. The CD spectra of (+)-**2b** and (−)-**2b** are mirror images of each other, and the CD spectrum of (+)-**2b** showed a large negative signal at 257 nm, a small negative signal at 292 nm, a large positive signal at 318 nm, a small negative signal at 335 nm, and broad positive signals around 345 and 415 nm. Based on the time-dependent-density functional theory (TD-DFT) calculation of a (*P*)-helix model of **2b** ((*P*)-**2c**) containing methyl groups instead of the isobutyl groups (Figure S3), the helicity of the (+)-**2b** was assigned to the (*P*)-helix. The maximum dissymmetric factor of absorption *g*_abs_ (Δε/ε) of (+)-**2b** was 5.9 × 10^–3^ at 422 nm. The CPL spectra of (+)-**2b** and (−)-**2b** in chloroform also displayed a mirror-image relationship, and the maximum luminescence dissymmetry factor *g*_lum_ of (+)-**2b** was 8.4 × 10^–3^ at 508 nm. This value is larger than that of carbo[7]­helicene^58^ and comparable to that of tetraaza[7]­helicene.^20^

**Figure 5:**
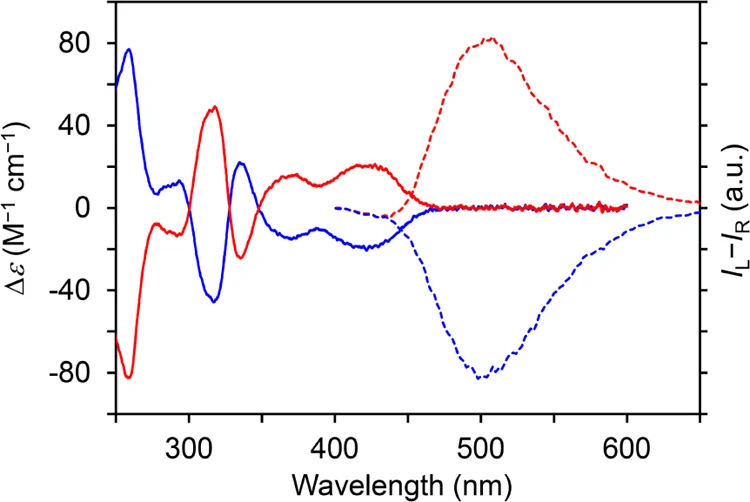
CD (solid lines) and CPL (dashed lines) spectra of (+)-**2b** (red) and (−)-**2b** (blue) in chloroform at room temperature. [**2b**] = 1.0 × 10^–5^ M.

## 3 Conclusions

We have developed a synthetic method to construct helicene structures with unidirectional amide linkages via the direct Pd-mediated intramolecular arylation of linear diamides and triamides, and the crystal structures and chiroptical properties of the resulting helicene analogues were elucidated. The direct intramolecular arylation of diamide **3** using Pd­(OAc)_2_ and PPh_3_ at 80 °C afforded [5]­helicene analogue **1** in 73% yield. The same reaction of triamide **4a** did not give [7]­helicene analogue **2a** because of a side reaction at the 3-position of the terminal 2-iodoaniline unit. The introduction of a methyl group at the 3-position suppressed the side reaction, and the intramolecular direct arylation of methylated triamide **4b** afforded the target [7]­helicene analogue **2b** in a yield of 29%. The X-ray diffraction analysis of a single crystal of **2b** revealed a helical structure. The fluorescence intensity of **2b** in dichloromethane was enhanced by the addition of 2000 equiv of TFA, whereas the absorption intensity was not affected. The enantiomers (+)-**2b** and (−)-**2b** were obtained by chiral resolution and were found to exhibit mirror image CD and CPL spectra with a *g*_lum_ value of (+)-**2b** of 8.4 × 10^–3^ at 508 nm. Studies on the synthesis of other helicene analogues using the direct arylation strategy are currently in progress in our laboratory.

## 4 Experimental Section

### 4.1 General Information

#### 4.1.1 Instruments

TLC analyses were carried out on aluminum sheets coated with Merck Silica gel 60 F_254_ (0.25 mm), and visualization was accomplished with UV light. Column chromatography was performed on neutral silica gel 60N (Kanto Chemical Co. Inc., 63–210 μm). Chiral resolution was performed using a JASCO HPLC system equipped with a Chiralpak IC columns (250 mm × 4.6 mm and 250 mm × 10 mm, 5 μm) and hexane/isopropyl alcohol/CH_2_Cl_2_ = 20/1/1 as the mobile phase. The melting points were determined on a Bibby Sterilin SMP3 and were uncorrected. The ^1^H and ^13^C NMR spectra were recorded on a JEOL ECA-500. The internal standards of the ^1^H and ^13^C NMR spectra were tetramethylsilane (0.00 ppm) and the midpoint of CDCl_3_ (77.0 ppm) in CDCl_3_, respectively, and the midpoints of DMSO (2.50 and 39.70 ppm) in DMSO-*d*_6_. High-resolution mass spectra were performed on a Thermo Scientific Q-Exactive with a hybrid Quadrupole-Orbitrap analyzer using ESI ionization. IR spectra were recorded on a JASCO FT/IR-470 plus. UV–vis spectra were recorded on a Shimadzu UV-2600i. Fluorescence spectra were recorded on a JASCO FP-6500. Photoluminescence quantum yields in solution were determined by a combination of an integrating sphere (Labsphere, 4P-GPS-030-SF), a monochromated xenon light source, and a cooled CCD spectrometer. The sensitivity of the spectrometer was corrected using a standard illuminant. CD spectra were recorded on a JASCO J-1500. The specific rotation values were measured with a JASCO DIP-1000 polarimeter. CPL spectra were obtained on a laboratory-made apparatus; the samples were excited by 365 nm light generated by a UVLED (OptoSupply, OSV1XME3E1S) with a U360 filter and a U340 filter (HOYA Optics). The emission light was collected at an angle of 180° and passed through a photoelastic modulator (PEM-90, HINDS Instruments) equipped with a wire-grid-type polarizer and focused into a Jovin-Yvon HR 320 monochromator. A cooled photomultiplier module (Hamamatsu, H7844) was attached to the monochromator to obtain the emission signals. A UV-39 glass filter (HOYA Optics) was placed before the monochromator to cut the excitation light. A current-sensitive preamplifier (5182, Signal Recovery) was used to amplify and separate the signal into AC and DC outputs, which were measured by a lock-in amplifier (SR830, Stanford Research Systems) and a digital multimeter (2000, Keithley), respectively. The luminescence dissymmetry factors, *g* values of CPL, *g*_lum_, are defined by eq 1

1 $$g_{lum}=2\left(I_{L}-I_{R}\right)/\left(I_{L}+I_{R}\right)$$

where *I*_L_ and *I*_R_ denote the intensities of the left and right CPL, respectively.^57^ The *g*_lum_ values were practically obtained using eq 2

2 $$g_{lum}=2\left(\Delta I/I\right)\times f$$

where Δ*I* and *I* are the observed amplitudes of the AC and DC signals, respectively. The calibration factor of this system, *f* (1.3), was obtained by measuring the reported *g*_lum_ value of tris­(3-trifluoroacetyl-(+)-camphorato)­europium­(III), [Eu­(+)-facam_3_], in dry DMSO, where *g*_lum_ = −0.78 at 595.2 nm.^59^ The wavelength dependence of *f* was considered constant within the wavelength range of the measurements. X-ray data were collected on a Rigaku XtaLAB P200 diffractometer with multilayer mirror monochromated Cu Kα (λ = 1.54187 Å) and Mo Kα (λ = 0.71075 Å) and a hybrid photon counting detector (PILATUS 200K). The crystal structure was solved by direct methods (SHELXT Version 2018/2)^60^ and refined by full-matrix least-squares SHELXL-2018/3.^61^ These data can be obtained free of charge from The Cambridge Crystallographic Data Centre via https://www.ccdc.cam.ac.uk/structures/.

#### 4.1.2 Materials

All starting materials and anhydrous solvents were purchased from Sigma-Aldrich (St. Louis, MO, USA), Fujifilm Wako Pure Chemical (Osaka, Japan), Tokyo Kasei (TCI, Tokyo, Japan), Nacalai Tesque (Kyoto, Japan), or Kanto Kagaku (Tokyo, Japan) and were used as received.

### 4.2 Synthetic Procedure

#### 4.2.1 Synthesis of **6**

Isobutyraldehyde (184 mg, 2.55 mmol) and **5** (300 mg, 1.03 mmol) were dissolved in 1,2-dichloroethane (3.8 mL), and the solution was stirred at room temperature under a nitrogen atmosphere. NaBH­(OAc)_3_ (655 mg, 3.09 mmol) was added, and the mixture was stirred at room temperature for 2.5 h. After addition of saturated aqueous NaHCO_3_, the mixture was extracted with AcOEt, and the organic layer was dried over anhydrous MgSO_4_. Purification with silica gel column chromatography (hexane/CH_2_Cl_2_ = 2/1) afforded **6** (342 mg, 96%) as a pale-yellow syrup. ^1^H NMR (500 MHz, CDCl_3_, 22 °C): δ 8.29 (s, 1H), 6.31 (s, 1H), 4.58 (t, *J* = 5.4 Hz, 1H), 3.82 (s, 3H), 3.02 (dd, *J* = 5.4 and 6.9 Hz, 2H), 2.55 (s, 3H), 1.96 (nonet, *J* = 6.7 Hz, 1H), 1.03 (d, *J* = 6.6 Hz, 6H); ^13^C NMR (126 MHz, CDCl_3_, 22 °C): δ 166.42, 149.86, 143.39, 141.93, 118.46, 112.13, 80.42, 51.45, 51.34, 27.83, 22.53, 20.37; MS (ESI^+^) calcd for C_13_H_18_INO_2_H 348.0455 [M + H]^+^, found 348.0457; calcd for C_13_H_18_INO_2_Na 370.0274 [M + Na]^+^, found 370.0272; calcd for C_13_H_18_INO_2_K 386.0014 [M + K]^+^, found, 386.0015; IR (KBr) 3397, 2955, 1712, 1589, 1433, 1323, 1243, 1087, 777 cm^–1^.

#### 4.2.2 Synthesis of **7**

^*i*^Pr_2_NEt (9.4 mL, 54 mmol) was added to a solution of **6** (5.00 g, 14.4 mmol) and benzoyl chloride (BzCl, 4.3 mL, 37 mmol) in dry CH_2_Cl_2_ (30 mL) at room temperature, and the mixture was stirred at room temperature for 28 h under a nitrogen atmosphere. After addition of 2 M hydrochloric acid, the mixture was extracted with AcOEt, and the organic layer was washed with water, saturated aqueous NaHCO_3_, and brine and dried over anhydrous MgSO_4_. Repeated purification with silica gel column chromatography (hexane/AcOEt = 15/1 then 4/1) afforded **7** (6.15 g, 96%) as a pale-yellow solid. mp 144.9–148.7 °C; ^1^H NMR (500 MHz, CDCl_3_, 22 °C): δ 8.28 (s, 1H), 7.33 (d, *J* = 7.4 Hz, 2H), 7.22 (t, *J* = 7.3 Hz, 1H), 7.15 (t, *J* = 7.0 Hz, 2H), 7.00 (s, 1H), 4.28 (dd, *J* = 9.2 and 13.7 Hz, 1H), 3.85 (s, 3H), 3.13 (dd, *J* = 5.4 and 13.5 Hz, 1H), 2.44 (s, 3H), 1.94 (nonet, *J* = 6.9 Hz, 1H), 1.07 (d, *J* = 6.6 Hz, 3H), 0.97 (d, *J* = 6.6 Hz, 3H); ^13^C NMR (126 MHz, CDCl_3_, 22 °C): δ 170.14, 165.43, 148.29, 142.18, 141.15, 135.99, 134.18, 129.70, 129.52, 127.83, 127.45, 95.34, 55.74, 52.02, 27.02, 21.15, 20.61, 20.27; MS (ESI^+^) calcd for C_20_H_22_INNaO_3_^+^ 474.0537 [M + Na]^+^, found 474.0535; IR (KBr) 3421, 2954, 1720, 1647, 1585, 1442, 1396, 1338, 1288, 1246, 1188, 1092, 914, 783, 702, 463 cm^–1^.

#### 4.2.3 Synthesis of **8**

KOH (ground in a mortar, 79.0 mg, 1.41 mmol) was added to a mixture of **7** (101 mg, 0.225 mmol) in CH_3_OH/H_2_O (1/1, 1.25 mL) at room temperature, and the mixture was stirred at reflux for 3 h and then cooled to room temperature. After addition of 1 M hydrochloric acid, the mixture was extracted with AcOEt, and the organic layer was dried over anhydrous MgSO_4_. Purification with silica gel column chromatography (hexane/AcOEt = 1/1, AcOEt, then CH_3_OH) afforded **8** (93.2 mg, 96%) as an orange solid. mp 207.0–208.7 °C; ^1^H NMR (500 MHz, CDCl_3_, 22 °C): δ 8.39 (s, 1H), 7.34 (d, *J* = 7.4 Hz, 2H), 7.23 (t, *J* = 7.2 Hz, 1H), 7.16 (t, *J* = 7.2 Hz, 2H), 7.01 (s, 1H), 4.29 (dd, *J* = 8.6 and 13.2 Hz, 1H), 3.15 (dd, *J* = 5.4 and 13.5 Hz, 1H), 2.47 (s, 3H), 1.95 (nonet, *J* = 6.3 Hz, 1H), 1.08 (d, *J* = 6.6 Hz, 3H), 0.98 (d, *J* = 6.6 Hz, 3H); ^13^C NMR (126 MHz, CDCl_3_, 21 °C): δ 170.69, 169.76, 148.72, 143.05, 141.89, 135.75, 134.33, 129.78, 129.39, 127.94, 127.60, 95.33, 55.96, 27.10, 21.51, 20.68, 20.34; MS (ESI^+^) calcd for C_19_H_21_INO_3_^+^ 438.0561 [M + H]^+^, found 438.0557; calcd for C_19_H_20_INNaO_3_^+^ 460.0380 [M + Na]^+^, found 460.0371; IR (KBr) 2970, 1724, 1620, 1408, 1292, 1218, 1130, 1088, 852, 698, 463 cm^–1^.

#### 4.2.4 Synthesis of **9**

Isobutyraldehyde (0.614 g, 8.51 mmol) and 2-iodoaniline (1.50 g, 6.85 mmol) were dissolved in 1,2-dichloroethane (30 mL), and the solution was stirred at room temperature under a nitrogen atmosphere. NaBH­(OAc)_3_ (2.02 g, 9.52 mmol) was added, and the mixture was stirred at room temperature for 2 h. Then, isobutyraldehyde (0.633 g, 8.78 mmol) and NaBH­(OAc)_3_ (2.02 g, 9.52 mmol) were added, and the mixture was stirred at room temperature for 1 h. After addition of saturated aqueous NaHCO_3_, the mixture was extracted with AcOEt, and the organic layer was dried over anhydrous MgSO_4_. Purification with silica gel column chromatography (hexane/CH_2_Cl_2_ = 40/1) afforded **9** (1.80 g, 96%) as an orange liquid. ^1^H NMR (500 MHz, CDCl_3_, 22 °C): δ 7.64 (dd, *J* = 1.4 and 7.7 Hz, 1H), 7.19 (td, *J* = 7.6 and 1.6 Hz, 1H), 6.54 (dd, *J* = 1.4 and 8.3 Hz, 1H), 6.41 (td, *J* = 7.4 and 1.5 Hz, 1H), 4.23 (br, 1H), 2.97 (dd, *J* = 5.7 and 6.6 Hz, 2H), 1.95 (nonet, *J* = 6.7 Hz, 1H), 1.01 (d, *J* = 6.6 Hz, 6H); ^13^C NMR (126 MHz, CDCl_3_, 21 °C): δ 147.33, 138.92, 129.36, 118.17, 110.48, 85.38, 51.90, 27.75, 20.46; MS (ESI^+^) calcd for C_10_H_15_IN^+^ 276.0244 [M + H]^+^, found 276.0239; IR (KBr) 3398, 2954, 1589, 1508, 1319, 1161, 1003, 741, 447 cm^–1^.

#### 4.2.5 Synthesis of **3**

A mixture of **8** (100 mg, 0.229 mmol) and thionyl chloride (1.0 mL, 14 mmol) was stirred at room temperature for 5 h and concentrated under reduced pressure. A solution of **9** (75.5 mg, 0.274 mmol) in dry CH_2_Cl_2_ (0.5 mL) was added dropwise at room temperature, and then ^*i*^Pr_2_NEt (0.10 mL, 0.57 mmol) was added at room temperature. The mixture was stirred at 35 °C for 23 h and cooled to room temperature. After addition of 2 M hydrochloric acid, the mixture was extracted with AcOEt, and the organic layer was washed with water, saturated aqueous NaHCO_3_, and brine and dried over anhydrous MgSO_4_. Purification with silica gel column chromatography (hexane/AcOEt = 4/1) afforded **3** (144 mg, 91%) as an orange solid. mp 162.7–163.5 °C; ^1^H NMR (500 MHz, DMSO-*d*_6_, 150 °C): δ 7.85 (br d, *J* = 7.2 Hz, 1H), 7.59 (br, 1H), 7.46–7.04 (m 8H), 6.99 (br, 1H), 3.96 (br, 2H), 3.19 (br, 2H), 2.31 (s, 3H), 1.84 (br, 2H), 0.90 (br, 12H); ^13^C NMR (126 MHz, DMSO-*d*_6_, 150 °C): δ 168.87, 166.80, 144.66, 143.40, 139.52, 136.73, 136.37, 136.06, 135.82, 132.52, 130.14, 128.79, 128.61, 128.30, 126.81, 98.55, 93.92, 55.89, 26.24, 19.96, 18.31; MS (ESI^+^) calcd for C_29_H_33_I_2_N_2_O_2_^+^ 695.0626 [M + H]^+^, found 695.0630; calcd for C_29_H_32_I_2_N_2_NaO_2_^+^ 717.0445 [M + Na]^+^, found 717.0446; IR (KBr) 3440, 3058, 2958, 1651, 1466, 1389, 1296, 1119, 1057, 1022, 895, 768, 702, 602, 455 cm^–1^.

#### 4.2.6 Typical Procedure of Intramolecular Direct Arylation of **3** for the Synthesis of **1** (Table 1)

To a round-bottomed flask were added **3** (100 mg, 0.154 mmol), Pd­(OAc)_2_ (6.9 mg, 0.031 mmol), PPh_3_ (16.2 mg, 0.0616 mmol), and K_2_CO_3_ (85.2 mg, 0.616 mmol), and the flask was degassed under reduced pressure and filled with argon. Then, dry DMF (1.6 mL) was added, and the flask was degassed under reduced pressure and filled with argon again. The mixture was refluxed for 2 h under an argon atmosphere, cooled to room temperature, and diluted with CH_2_Cl_2_. The mixture was filtered with Celite, and the filtrate was washed with brine and dried over MgSO_4_. Purification with silica gel column chromatography (hexane/AcOEt = 4/1) afforded **1** (37.1 mg, 55%) as a yellow solid and a mixture of products. The mixture was further separated with silica gel column chromatography (CH_2_Cl_2_/CH_3_OH = 50/1) to give **10** (16.8 mg, 14%) as a yellow solid.

**1**: mp 177.8–181.2 °C; ^1^H NMR (500 MHz, CDCl_3_, 21 °C): δ 8.44 (dd, *J* = 1.4 and 8.0 Hz, 1H), 7.97 (d, *J* = 8.3 Hz, 1H), 7.88 (d, *J* = 7.4 Hz, 1H), 7.46 (ddd, *J* = 1.1, 7.2, and 8.0 Hz, 1H), 7.41–7.33 (m, 3H), 7.22 (s, 1H), 6.87 (ddd, *J* = 1.4, 6.6, and 8.0 Hz, 1H), 4.67–4.53 (m, 1H), 4.51–4.37 (m, 1H), 4.31–4.17 (m, 1H), 4.00–3.91 (m, 1H), 2.98 (s, 3H), 2.41–2.27 (m, 2H), 1.12 (d, *J* = 6.6 Hz, 3H), 1.09 (d, *J* = 6.9 Hz, 3H), 1.02 (d, *J* = 6.9 Hz, 3H), 0.99 (d, *J* = 6.6 Hz, 3H); ^13^C NMR (126 MHz, CDCl_3_, 21 °C): δ 162.31, 161.97, 142.51, 140.56, 136.95, 135.15, 134.13, 130.80, 130.31, 129.48, 128.18, 127.93, 127.57, 125.66, 121.43, 120.57, 120.19, 118.28, 115.04, 112.95, 49.17, 48.13, 31.53, 27.49, 27.01, 24.71, 20.47, 20.17, 19.90; MS (ESI^+^) calcd for C_29_H_31_N_2_O_2_^+^ 439.2381 [M + H]^+^, found 439.2367; IR (KBr) 3437, 2958, 1651, 1597, 1462, 1331, 1300, 1211, 1111, 1041, 806, 752, 613, 525 cm^–1^.

**10**: mp 176.8–178.4 °C; ^1^H NMR (500 MHz, CDCl_3_, 22 °C): δ 7.72–7.67 (m, 6H), 7.42–7.36 (m, 4H), 7.30–7.27 (m, 8H), 6.95–6.93 (m, 3H), 6.79 (d, *J* = 8.0 Hz, 1H), 6.64 (dd, *J* = 7.5 and 8.3 Hz, 1H), 6.44 (dd, *J* = 1.4 and 7.2 Hz, 1H), 4.12 (br, 2H), 3.88 (br, 2H), 2.91 (s, 3H), 2.34–2.23 (m, 1H), 2.08–1.98 (m, 1H), 1.00 (d, *J* = 6.9 Hz, 6H), 0.70 (d, *J* = 6.6 Hz, 6H); ^13^C NMR (126 MHz, CDCl_3_, 22 °C): δ 169.92, 163.49, 156.70, 156.44, 156.35, 153.18, 139.95, 139.14, 137.80, 136.91, 135.08 (d, *J*_PC_ = 13.2 Hz), 134.61, 133.85 (d, *J*_PC_ = 9.0 Hz), 132.61, 132.35, 130.11, 129.90 (d, *J*_PC_ = 1.8 Hz), 128.31, 128.24, 128.13 (d, *J*_PC_ = 18.0 Hz), 128.05, 126.40, 126.38, 119.06, 118.40, 118.35, 109.81, 58.75, 48.86, 27.47, 27.27, 23.75, 20.35, 19.58; MS (ESI^+^) calcd for C_47_H_46_N_2_O_2_PPd^+^ 807.2332 [M + H]^+^, found 807.2341; IR (KBr) 3453, 2959, 2360, 1636, 1593, 1538, 1411, 1261, 1095, 800, 744, 696, 521 cm^–1^.

#### 4.2.7 Synthesis of **11**

A mixture of **8** (4.00 g, 9.15 mmol) and thionyl chloride (40 mL, 0.55 mol) was stirred at room temperature for 25 h and concentrated under reduced pressure. A solution of **6** (3.49 g, 10.1 mmol) in dry CH_2_Cl_2_ (20 mL) was added dropwise at room temperature, and then ^*i*^Pr_2_NEt (4.0 mL, 23 mmol) was added at room temperature. The mixture was stirred at room temperature for 24 h and then 2 M hydrochloric acid was added. The mixture was extracted with AcOEt, and the organic layer was washed with water, saturated aqueous NaHCO_3_, and brine and dried over anhydrous MgSO_4_. Purification with silica gel column chromatography (hexane/AcOEt = 3/1 then AcOEt) afforded **11** (6.39 g, 91%) as an orange solid. mp 197.4–198.1 °C; ^1^H NMR (500 MHz, DMSO-*d*_6_, 150 °C): δ 8.17–8.14 (m, 1H), 7.84–7.00 (m, 8H), 4.46–3.50 (m 5H), 3.23 (br, 2H), 2.43–2.40 (m, 3H), 2.32 (s, 3H), 1.83 (br, 2H), 0.97–0.90 (m, 12H); ^13^C NMR (126 MHz, DMSO-*d*_6_, 150 °C): δ 168.87, 166.66, 164.77, 146.41, 144.83, 140.59, 140.48, 139.52, 137.05, 136.08, 133.04, 132.55, 130.17, 128.55, 126.95, 126.87, 126.69, 94.35, 55.89, 51.32, 26.38, 26.27, 19.98, 19.91, 19.20, 18.37; MS (ESI^+^) calcd for C_32_H_36_I_2_N_2_NaO_4_^+^ 789.0657 [M + Na]^+^, found 789.0663; IR (KBr) 2958, 1724, 1651, 1589, 1442, 1389, 1335, 1284, 1092, 903, 895, 783, 702, 602 cm^–1^.

#### 4.2.8 Synthesis of **12**

KOH (ground in a mortar, 1.86 g, 33.2 mmol) was added to a mixture of **11** (4.00 g, 5.22 mmol) in CH_3_OH/H_2_O (1/1, 76 mL) at room temperature, and the mixture was stirred at reflux for 24 h and then cooled to room temperature. After addition of 1 M hydrochloric acid, the mixture was extracted with AcOEt, and the organic layer was dried over anhydrous MgSO_4_. Purification with silica gel column chromatography (hexane/AcOEt = 1/1 then AcOEt) afforded **12** (3.91 g, quant) as an orange solid. mp 219.8–222.3 °C; ^1^H NMR (500 MHz, DMSO-*d*_6_, 150 °C): δ 8.19–8.15 (m, 1H), 7.88–6.82 (m, 8H), 3.94 (br, 2H), 3.24 (br, 2H), 2.42 (br s, 3H), 2.32 (s, 3H), 1.84 (br, 2H), 0.89 (br, 12H); ^13^C NMR (126 MHz, DMSO-*d*_6_, 150 °C): δ 168.89, 166.71, 165.67, 145.96, 144.80, 140.78, 139.51, 137.05, 136.07, 135.93, 132.98, 132.54, 131.33, 128.60, 126.86, 126.71, 94.17, 55.87, 26.38, 26.28, 19.95, 19.47, 18.38; MS (ESI^+^) calcd for C_31_H_35_I_2_N_2_O_4_^+^ 753.0681 [M + H]^+^, found 753.0681; calcd for C_31_H_34_I_2_N_2_NaO_4_^+^ 775.0500 [M + Na]^+^, found 775.0497; IR (KBr) 2962, 1720, 1651, 1446, 1392, 1335, 1288, 1122, 903, 787, 702, 606, 459 cm^–1^.

#### 4.2.9 Synthesis of **4a**

A mixture of **12** (501 mg, 0.666 mmol) and thionyl chloride (3.0 mL, 41 mmol) was stirred at room temperature for 28 h and concentrated under reduced pressure. A solution of **9** (203 mg, 0.738 mmol) in dry CH_2_Cl_2_ (2.5 mL) was added dropwise at room temperature, and then ^*i*^Pr_2_NEt (0.3 mL, 2 mmol) was added at room temperature. The mixture was stirred at room temperature for 67 h under a nitrogen atmosphere, and then 2 M hydrochloric acid was added. The mixture was extracted with AcOEt, and the organic layer was washed with water, saturated aqueous NaHCO_3_, and brine and dried over anhydrous MgSO_4_. Purification with silica gel column chromatography (hexane/AcOEt = 2/1) afforded **4a** (509 mg, 76%) as an orange solid. mp 232.2–233.1 °C; ^1^H NMR (500 MHz, DMSO-*d*_6_, 150 °C): δ 7.88 (br d, *J* = 7.7 Hz, 1H), 7.64 (br, 2H), 7.46–6.71 (m, 10H), 4.62–2.92 (m, 6H), 2.31 (m, 3H), 2.27 (m, 3H), 2.06–1.43 (m, 3H), 1.13–0.46 (m, 18H). We were unable to obtain an analyzable ^13^C NMR spectrum (126 MHz, DMSO-*d*_6_, 150 °C) of **4a** probably due to the slow interconversion between the conformers even at 150 °C indicated by broad signals of ^1^H NMR spectrum at 150 °C; MS (ESI^+^) calcd for C_41_H_47_I_3_N_3_O_3_^+^, 1010.0746 [M + H]^+^, found 1010.0750; calcd for C_41_H_46_I_3_N_3_NaO_3_^+^ 1032.0565 [M + Na]^+^, found 1032.0557; IR (KBr) 3448, 3059, 2962, 2873, 1655, 1585, 1466, 1389, 1335, 1288, 1119, 1061, 1022, 895, 768, 721, 598, 455 cm^–1^.

#### 4.2.10 Intramolecular Direct Arylation of **4a**

To a round-bottomed flask, **4a** (100 mg, 0.0991 mmol), Pd­(OAc)_2_ (13.8 mg, 0.0615 mmol), PPh_3_ (31.3 mg, 0.119 mmol), and K_2_CO_3_ (82.5 mg, 0.597 mmol) were placed, and the flask was degassed under reduced pressure and filled with argon. Then, dry DMF (1.6 mL) was added, and the flask was degassed under reduced pressure and filled with argon again. The mixture was stirred at 80 °C for 48 h under an argon atmosphere, cooled to room temperature, and diluted with CH_2_Cl_2_. The mixture was filtered with Celite, and the filtrate was washed with brine and dried over MgSO_4_. Twice purification with silica gel column chromatography (hexane/AcOEt = 4/1) afforded **13** (10.4 mg, 17%) as a yellow solid. mp: 250.6–252.5 °C; ^1^H NMR (500 MHz, CDCl_3_, 21 °C): δ 8.87 (d, *J* = 8.3 Hz, 1H), 7.92 (t, *J* = 8.3 Hz, 1H), 7.65 (d, *J* = 8.0 Hz, 1H), 7.60 (s, 1H), 7.35 (dd, *J* = 1.1 and 8.3 Hz, 2H), 7.21 (d, *J* = 0.6 Hz, 1H), 7.16 (t, *J* = 7.4 Hz, 1H), 7.04 (t, *J* = 7.9 Hz, 2H), 4.62–4.26 (m, 5H), 3.27 (s, 3H), 2.99 (dd, *J* = 5.4 and 13.7 Hz, 1H), 2.90 (s, 3H), 2.50–2.38 (m, 2H), 1.83–1.74 (m, 1H), 1.12 (d, *J* = 6.6 Hz, 6H), 1.10–1.07 (m, 9H), 0.69 (d, *J* = 6.6 Hz, 3H); ^13^C NMR (126 MHz, CDCl_3_, 22 °C): δ 170.50, 162.40, 162.02, 143.92, 142.91, 142.70, 138.62, 138.03, 136.01, 135.92, 131.07, 130.03, 129.27, 129.18, 128.64, 127.91, 127.86, 121.32, 120.80, 118.79, 116.90, 116.17, 115.70, 112.97, 110.55, 55.45, 49.11, 42.64, 27.59, 27.15, 26.91, 25.97, 24.63, 20.80, 20.75, 20.32, 20.31, 20.29, 20.28; MS (ESI^+^) calcd for C_41_H_44_N_3_O_3_^+^ 626.3383 [M + H]^+^, found 626.3373, calcd for C_41_H_43_N_3_NaO_3_^+^ 648.3197 [M + Na]^+^, found 648.3191; IR (KBr) 3443, 2960, 2360, 1653, 1603, 1448, 1375, 1334, 757 cm^–1^.

#### 4.2.11 Synthesis of **14**

Isobutyraldehyde (38.8 mg, 0.538 mmol) and 2-iodo-3-methylaniline^62^ (50.2 mg, 0.215 mmol) were dissolved in 1,2-dichloroethane (0.65 mL), and the solution was stirred at room temperature under a nitrogen atmosphere. NaBH­(OAc)_3_ (137 mg, 0.646 mmol) was added, and the mixture was stirred at room temperature for 2 h. After addition of saturated aqueous NaHCO_3_, the mixture was extracted with AcOEt, and the organic layer was dried over anhydrous Na_2_SO_4_. Purification with silica gel column chromatography (hexane/AcOEt = 16/1) afforded **14** (58.9 mg, 95%) as a yellow liquid. ^1^H NMR (500 MHz, CDCl_3_, 22 °C): δ 7.07 (t, *J* = 7.7 Hz, 1H), 6.61 (ddd, *J* = 0.6, 1.4, and 7.4 Hz, 1H), 6.37 (d, *J* = 8.0 Hz, 1H), 4.44 (br s, 1H), 2.97 (dd, *J* = 5.4 and 6.6 Hz, 2H), 2.42 (s, 3H), 1.96 (nonet, *J* = 6.6 Hz, 1H), 1.02 (d, *J* = 6.6 Hz, 6H); ^13^C NMR (126 MHz, CDCl_3_, 23 °C): δ 147.59, 142.17, 128.61, 118.21, 107.70, 92.71, 52.27, 29.65, 27.75, 20.50; MS (ESI^+^) calcd for C_11_H_17_IN^+^ 290.0400 [M + H]^+^, found 290.0404; IR (KBr) 3398, 2955, 1587, 1498, 1465, 1322, 1153, 1001, 761, 500 cm^–1^.

#### 4.2.12 Synthesis of **4b**

A mixture of **12** (500 mg, 0.665 mmol) and thionyl chloride (3.0 mL, 41 mmol) was stirred at room temperature for 24 h and concentrated under reduced pressure. A solution of **14** (213 mg, 0.738 mmol) in dry CH_2_Cl_2_ (3.0 mL) was added dropwise at room temperature, and then ^*i*^Pr_2_NEt (0.3 mL, 2 mmol) was added at room temperature. The mixture was stirred at room temperature for 64 h under a nitrogen atmosphere, and then 2 M hydrochloric acid was added. The mixture was extracted with AcOEt, and the organic layer was washed with water, saturated aqueous NaHCO_3_, and brine and dried over anhydrous MgSO_4_. Purification with silica gel column chromatography (hexane/AcOEt = 2/1 then AcOEt) afforded **4b** (482 mg, 71%) as an orange solid. mp: 230.7–233.5 °C; ^1^H NMR (500 MHz, DMSO-*d*_6_, 150 °C): δ 7.92–6.66 (m, 12H), 4.44–2.94 (m, 6H), 2.49–2.08 (m, 9H), 2.08–1.33 (m, 3H), 1.09–0.49 (m, 18H). We were unable to obtain an analyzable ^13^C NMR spectrum (126 MHz, DMSO-*d*_6_, 150 °C) of **4b** probably due to the slow interconversion between the conformers even at 150 °C indicated by broad signals of ^1^H NMR spectrum at 150 °C; MS (ESI^+^) calcd for C_42_H_49_I_3_N_3_O_3_^+^ 1024.0902 [M + H]^+^, found 1024.0878; calcd for C_42_H_48_I_3_N_3_NaO_3_^+^ 1046.0722 [M + Na]^+^, found 1046.0682; IR (KBr) 3490, 2960, 1655, 1578, 1463, 1393, 1335, 1291, 1120, 1057, 896, 700 cm^–1^.

#### 4.2.13 Typical Procedure of Intramolecular Direct Arylation of **4b** for the Synthesis of **2b** (Table 2)

To a round-bottomed flask, **4b** (50.0 mg, 0.0498 mmol), Pd­(OAc)_2_ (6.6 mg, 0.029 mmol), PPh_3_ (15.4 mg, 0.058 mmol), and K_2_CO_3_ (40.5 mg, 0.293 mmol) were placed, and the flask was degassed under reduced pressure and filled with argon. Then, dry DMF (9.6 mL) was added, and the flask was degassed under reduced pressure and filled with argon again. The mixture was stirred at 80 °C for 62 h under an argon atmosphere, cooled to room temperature, and diluted with CH_2_Cl_2_. The mixture was filtered with Celite, and the filtrate was washed with brine and dried over MgSO_4_. Twice purification with silica gel column chromatography (hexane/AcOEt = 4/1) afforded **2b** (8.0 mg, 26%) as a yellow solid and **15** (14.7 mg, 47%) as an orange solid.

**2b**: mp 272.8–275.0 °C; ^1^H NMR (500 MHz, CDCl_3_, 22 °C): δ 7.94 (dd, *J* = 1.2 and 8.0 Hz, 1H), 7.28 (s, 1H), 7.17 (s, 1H), 7.14 (ddd, *J* = 0.9, 7.2, and 8.0 Hz, 1H), 6.96 (ddd, *J* = 1.4, 7.2, and 8.3 Hz 1H), 6.92 (dd, *J* = 7.5 and 8.3 Hz, 1H), 6.80 (d, *J* = 7.7 Hz, 1H), 6.44 (d, *J* = 8.3 Hz, 1H), 6.31 (d, *J* = 7.2 Hz, 1H), 4.65 (br, 1H), 4.20 (br, 2H), 4.04 (br, 1H), 3.75 (br, 2H), 3.07 (s, 3H), 3.04 (s, 3H), 2.45–2.36 (m, 1H), 2.28 (nonet, *J* = 6.9 Hz, 1H), 1.98 (nonet, *J* = 6.9 Hz, 1H), 1.17 (s, 3H), 1.15 (d, *J* = 6.9 Hz, 3H), 1.13 (d, *J* = 6.9 Hz, 3H), 1.02–0.99 (m, 12H); ^13^C NMR (126 MHz, CDCl_3_, 23 °C): δ 162.46, 161.92, 161.62, 143.21, 142.58, 139.59, 138.69, 137.57, 136.98, 134.42, 133.68, 133.22, 129.67, 128.20, 127.71, 126.32, 124.55, 123.47, 123.44, 122.00, 121.68, 119.40, 117.73, 116.92, 115.16, 113.66, 111.36, 49.44, 49.16, 48.54, 27.86, 27.37, 27.24, 24.87, 24.63, 20.99, 20.49, 20.41, 20.39, 20.34, 20.30, 19.99; MS (ESI^+^) calcd for C_42_H_46_N_3_O_3_^+^ 640.3534 [M + H]^+^, found 640.3555, calcd for C_42_H_45_N_3_NaO_3_^+^ 662.3353 [M + Na]^+^, found 662.3373; IR (KBr) 3346, 2960, 2361, 1649, 1590, 1460, 1263, 1081, 804 cm^–1^.

**15**: mp 241.6–244.5 °C; ^1^H NMR (500 MHz, DMSO-*d*_6_, 120 °C): δ 8.24 (d, *J* = 7.7 Hz, 1H), 8.10 (s, 1H), 7.99 (br, 1H), 7.55 (t, *J* = 8.0 Hz, 1H), 7.46 (t, *J* = 7.7 Hz, 2H), 7.34 (d, *J* = 7.5 Hz, 1H), 7.30 (s, 1H), 7.27 (s, 1H), 7.16 (d, *J* = 8.0 Hz, 1H), 4.20 (s, 2H), 4.16 (d, *J* = 7.5 Hz, 2H), 3.95 (d, *J* = 7.2 Hz, 2H), 3.93 (d, *J* = 6.6 Hz, 2H), 2.74 (s, 3H), 2.61 (s, 3H), 2.12 (nonet, *J* = 6.8 Hz, 2H), 1.95–1.84 (m, 1H), 0.96 (d, *J* = 6.6 Hz, 6H), 0.85–0.83 (m, 12H); ^13^C NMR (126 MHz, DMSO-*d*_6_, 120 °C): δ 168.56, 161.69, 160.38, 142.79, 141.04, 140.93, 139.32, 136.86, 136.63, 135.16, 133.43, 132.25, 131.82, 130.15, 129.37, 129.25, 127.69, 127.21, 124.37, 122.25, 121.02, 117.81, 117.36, 116.53, 115.13, 110.67, 55.20, 48.16, 47.83, 36.17, 26.84, 26.46, 26.12, 19.78, 19.38, 19.24, 19.15; MS (ESI^+^) calcd for C_42_H_46_N_3_O_3_^+^ 640.3534 [M + H]^+^, found 640.3534, calcd for C_42_H_45_N_3_NaO_3_^+^ 662.3353 [M + Na]^+^, found 662.3354; IR (KBr) 3444, 2959, 1653, 1582, 1460, 1389, 1334, 1275, 1126, 1034, 897, 772 cm^–1^.

## Supplementary Material













## Data Availability

The data underlying this study are available in the published article and its Supporting Information.

## Notes

Crystallographic data for **1** (CIF)
Crystallographic data for **10** (CIF)
Crystallographic data for **13** (CIF)
Crystallographic data for **15** (CIF)
Crystallographic data for **2b** (CIF)
X-ray crystallographic data, concentration and solvent effects on absorption and fluorescence spectra of **2b**, copies of ^1^H and ^13^C NMR spectra, and DFT calculations (PDF)
