# Supplementary material for: A Direct Arylation Approach for Helicene Analogues Composed of Unidirectional Amide Linkages
Source: ACS Omega. 2026 Aug 20;11(34):52003–13. doi: 10.1021/acsomega.6c06995 (PMC13625081; doi:10.1021/acsomega.6c06995)

Supporting Information

A Direct Arylation Approach for Helicene Analogues Composed of Unidirectional Amide Linkages

Akihiro Yokoyama,*[a] Hirotaka Shimoda,[a] Shoko Kikkawa,[b] Isao Azumaya,[b] Taro Tsubomura,[a] and Kosuke Oki[a]

[a] Faculty of Science and Technology, Seikei University, 3-3-1 Kichijoji-kitamachi, Musashino, Tokyo 180-8633, Japan

[b] Faculty of Pharmaceutical Sciences, Toho University, 2-2-1 Miyama, Funabashi, Chiba 274-8510, Japan

E-mail: ayokoyama@st.seikei.ac.jp

Table of Contents

1. X-ray Crystallographic Data 2

2. Concentration and Solvent Effects on Absorption and Fluorescence Spectra of **2b** 4

3. Theoretical Study on the CD Spectrum of **2b** 5

4. References 7

5. ^{1}H and ^{13}C NMR Spectral Data 8

1. X-ray Crystallographic Data

All non-hydrogen atoms were refined anisotropically. A part of the C–H hydrogen atoms were located by difference Fourier synthesis: those in the helicene rings (**2b**), aromatic rings and C39 (**15**). The other hydrogens (aromatic and methyl hydrogens) were located on the calculated positions.

**Compounds**	**1**	**2b**
Formula	$C_{29}H_{30}N_2O_2$	$C_{42}H_{45}N_3O_3$
M_r	438.57	639.84
Crystal dimensions / mm^3	0.200 × 0.053 × 0.015	0.114 × 0.090 × 0.077
Crystal system	triclinic	monoclinic
Space group	$P\bar{1}$	$P2_1/n$
a / Å	11.68683(10)	16.87258(14)
b / Å	14.38318(11)	9.47499(5)
c / Å	15.28350(11)	21.03046(14)
α / deg	100.0849(6)	90
β / deg	106.5249(7)	90.9498(6)
γ / deg	106.1249(7)	90
V / Å^3	2274.21(4)	3361.63(4)
$\rho_{calcd.}$ / g•cm^{-3}	1.281	1.264
Z	4	4
Z'	2	1
Temperature / K	93	93
Goodness-of-fit on F^2	1.062	1.059
R_1 [$I > 2\sigma(I)$] on F	0.0376	0.0439
wR_2 (all data) on F^2	0.1115	0.1188
No. reflection collected (all data)	60154	43695
No. of independent reflections [$I > 2\sigma(I)$]	6749	5558
R_{int}	0.0354	0.1355
Abs. corr.	multi-scan	Lorentz-polarization
Radiation type	Cu$K\alpha$ (λ = 1.54187 Å)	Cu$K\alpha$ (λ = 1.54187 Å)
T_{max}	0.844	0.953
T_{min}	0.991	0.953
$2\theta_{max}$	136.5	136.5
μ /mm^{-1}	0.631	0.624
Largest diff. peak/hole / eÅ^{-3}	0.26 / -0.24	0.23 / -0.27
No. of parameters	605	478
CCDC No.	2554086	2554087

**Compound**	**10**	**13**	**15**
Formula	$C_{47}H_{45}N_2O_2PPd$	$C_{42}H_{45}Cl_2N_3O_3$	$C_{42}H_{45.57}N_3O_{3.29}$
M_r	807.26	710.74	645.05
Crystal dimensions / mm^3	0.311 × 0.232 × 0.099	0.279 × 0.166 × 0.077	0.100 × 0.090 × 0.050
Crystal system	triclinic	triclinic	triclinic
Space group	$P\bar{1}$	$P\bar{1}$	$P\bar{1}$
a / Å	12.2245(2)	10.0527(2)	8.0403(4)
b / Å	12.3862(2)	12.1778(2)	13.9713(3)
c / Å	14.7885(2)	15.61498(17)	15.9672(6)
α / deg	70.2282(15)	92.3250(14)	91.088(2)
β / deg	65.5978(16)	108.5455(15)	101.894(3)
γ / deg	78.9065(14)	95.9236(16)	97.620(3)
V / Å^3	1915.33(6)	1797.17(5)	1737.66(12)
$\rho_{calcd.}$ / g•cm^{-3}	1.400	1.313	1.233
Z	2	2	2
Z'	1	1	1
Temperature / K	93	93	93
Goodness-of-fit on F^2	1.076	1.043	1.053
R_1 [$I > 2\sigma(I)$] on F	0.0269	0.0478	0.0676
wR_2 (all data) on F^2	0.0738	0.1345	0.2076
No. reflection collected (all data)	62195	45853	41388
No. of independent reflections [$I > 2\sigma(I)$]	8187	6130	4693
R_{int}	0.0546	0.0616	0.0556
Abs. corr.	multi-scan	multi-scan	multi-scan
Radiation type	Mo$K\alpha$ (λ = 0.71075 Å)	Cu$K\alpha$ (λ = 1.54187 Å)	Cu$K\alpha$ (λ = 1.54187 Å)
T_{max}	0.624	0.643	0.851
T_{min}	0.945	0.859	0.970
$2\theta_{max}$	27.498	68.250	68.244
μ /mm^{-1}	0.569	1.972	0.614
Largest diff. peak/hole / eÅ^{-3}	0.75 / -0.87	0.39 / -0.58	0.42 / -0.33
No. of parameters	483	459	496
CCDC No.	2554088	2554089	2554090

2. Concentration and Solvent Effects on Absorption and Fluorescence Spectra of 2b

Effect of Concentration.

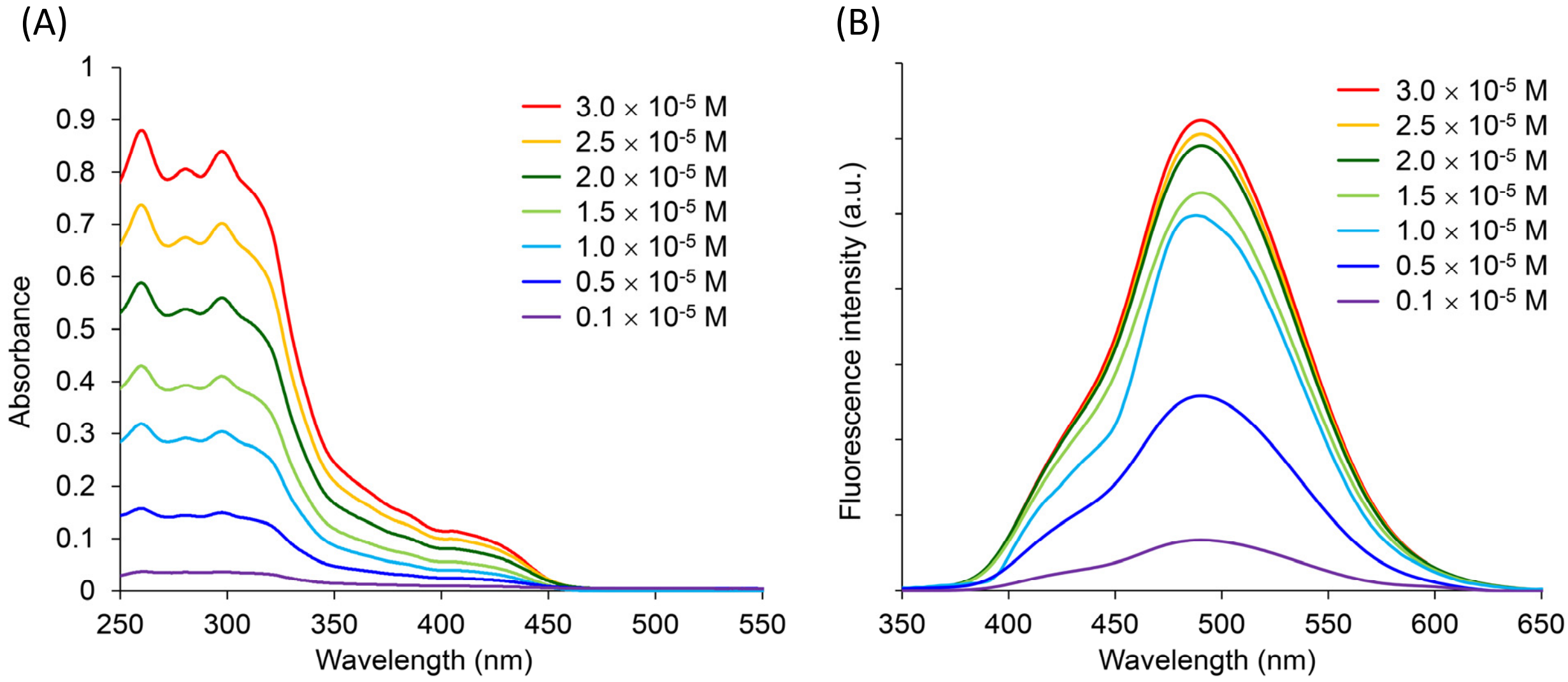

Figure S1. (A) Absorption and (B) fluorescence (λ_{ex} = 298 nm) spectra of **2b** in chloroform at room temperature. The concentration of **2b** was 0.1 × 10^{-5} M (purple line), 0.5 × 10^{-5} M (blue line), 1.0 × 10^{-5} M (light blue line), 1.5 × 10^{-5} M (yellow-green line), 2.0 × 10^{-5} M (green line), 2.5 × 10^{-5} M (yellow line), and 3.0 × 10^{-5} M (red line).

Effect of Solvent.

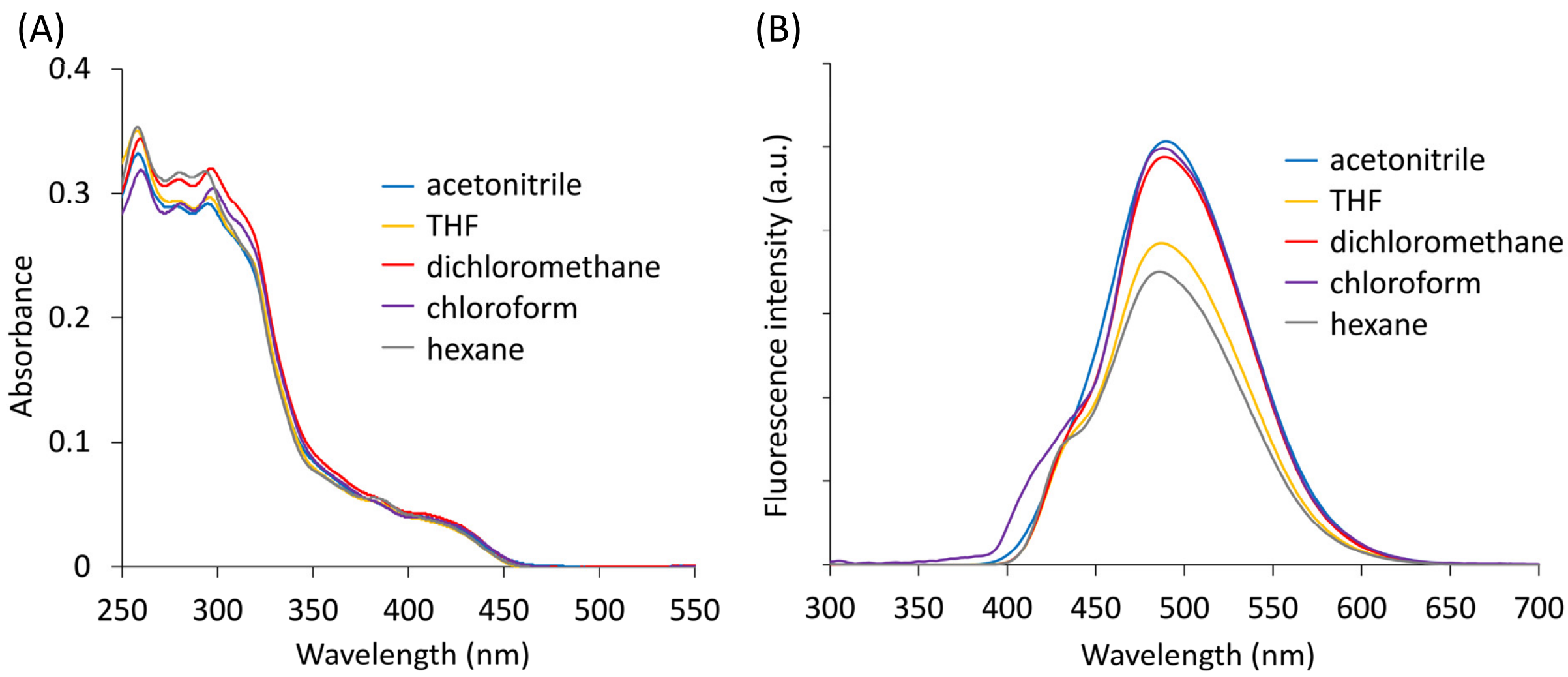

Figure S2. (A) Absorption and (B) fluorescence spectra of **2b** in acetonitrile (blue line, λ_{ex} = 296 nm), THF (yellow line, λ_{ex} = 296 nm), dichloromethane (red line, λ_{ex} = 296 nm), chloroform (purple line, λ_{ex} = 298 nm), and hexane (gray line, λ_{ex} = 298 nm) at room temperature ([**2b**] = 1.0 × 10^{-5} M).

3. Theoretical Study on the CD Spectrum of 2b

The (*P*)-helix model of **2b** ((*P*)-**2c**) containing methyl groups instead of isobutyl groups on the nitrogen atoms of amide bonds was used for the computational study for simplicity. The initial structure of (*P*)-**2c** was constructed on a Windows 10 PC with the GaussView 6 (Gaussian, Inc., Pittsburgh, PA). The structures were fully optimized by the density functional theory (DFT) calculations using the B3LYP[S1,2] functional with the 6-311G(2d,p) basis sets[S3] in Gaussian 16 software[S4]. Vibrational frequency calculation was then performed at the same level of theory to confirm that the optimized structure corresponds to minima (no imaginary frequencies). The theoretical CD spectrum of (*P*)-**2c** was calculated by the time-dependent-DFT (TD-DFT) at B3LYP/6-311G(2d,p) level of theory (Figure S3). The calculated rotational strengths in length gauge were expanded by the Gaussian functions and overlapped where the width of the band at the half height was fixed at 0.15 eV.

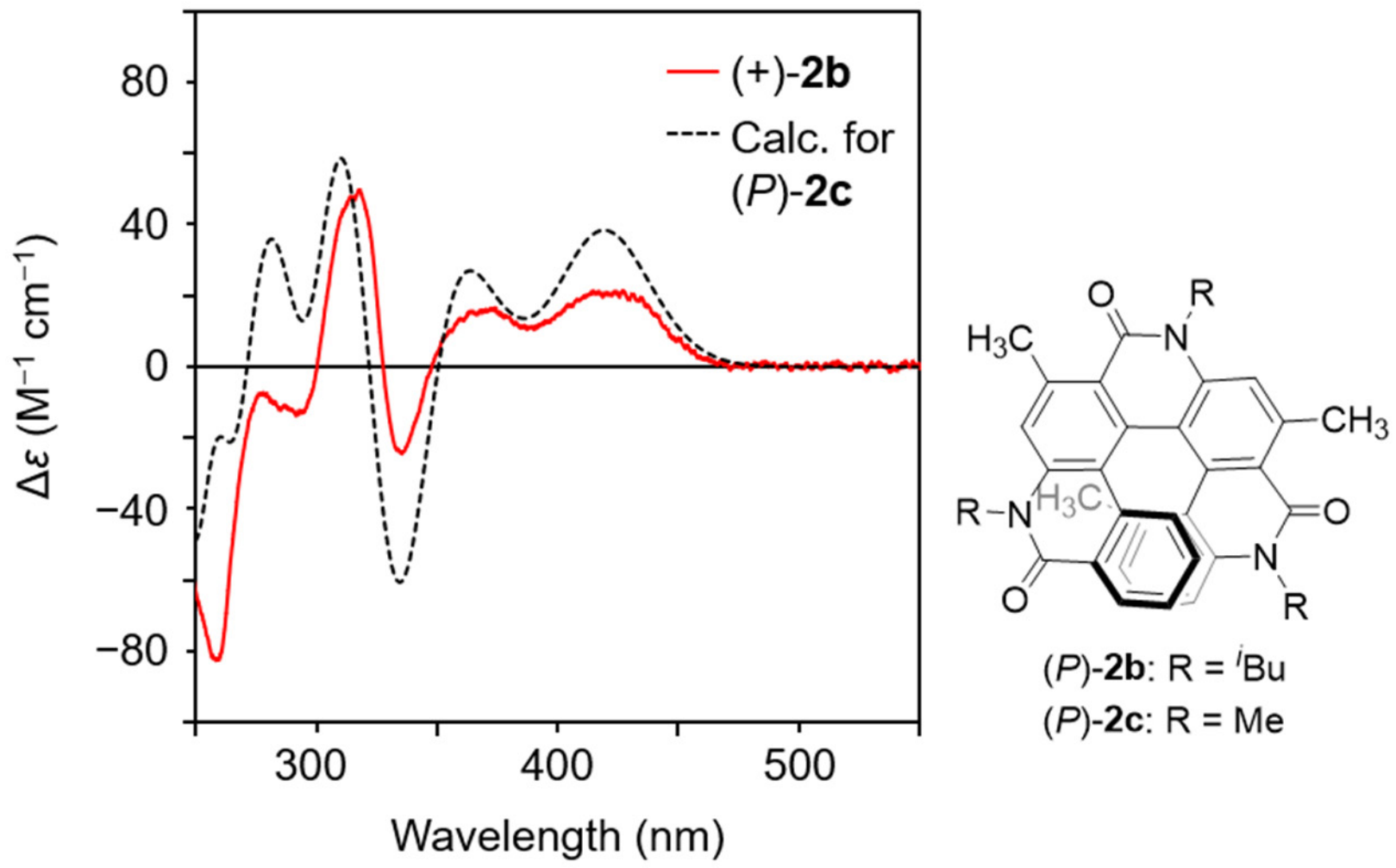

Figure S3. Experimental (red solid line) and calculated (black dashed line) CD spectra of (+)-**2b** in chloroform at room temperature ([**2b**] = 1.0 × 10^{-5} M) and (*P*)-**2c**, respectively.

The DFT Optimized Coordinate of 2c

Tag	Symbol	X	Y	Z
1	O	1.759048	-4.607918	-0.578700
2	O	-4.782046	1.071053	-0.155049
3	O	3.307023	3.831943	0.061495
4	N	-3.496466	-0.703594	0.437820
5	N	1.094296	3.618041	-0.406291
6	N	2.728132	-2.556716	-0.312506
7	C	1.668209	-3.394389	-0.673267
8	C	0.501456	-2.718492	-1.263469
9	C	-0.425540	-3.516773	-1.943058
10	H	-0.286035	-4.588993	-1.910658
11	C	-1.453063	-2.936618	-2.657836
12	H	-2.163470	-3.552726	-3.195473
13	C	-1.539423	-1.542664	-2.717963
14	H	-2.308574	-1.073447	-3.320148
15	C	-0.644474	-0.749618	-2.026722
16	H	-0.722435	0.323638	-2.110070
17	C	0.375208	-1.318868	-1.240719
18	C	1.385107	-0.534172	-0.528792
19	C	1.267426	0.840313	-0.178363
20	C	-0.019985	1.514732	0.016848
21	C	-1.215740	0.891181	0.473080
22	C	-1.241269	-0.348867	1.260122
23	C	-0.271313	-0.699857	2.231356
24	C	-0.342902	-1.951039	2.837102
25	H	0.403409	-2.218246	3.576243
26	C	-1.369071	-2.837823	2.544397
27	H	-1.392542	-3.816749	3.007658
28	C	-2.407657	-2.444723	1.721629
29	H	-3.250112	-3.102554	1.574178
30	C	-2.380938	-1.178944	1.125652
31	C	-3.678297	0.653016	0.170875
32	C	-2.458713	1.488057	0.175010
33	C	-2.515315	2.835434	-0.260782
34	C	-1.325060	3.527907	-0.387718
35	H	-1.368397	4.579060	-0.628178
36	C	-0.080310	2.893720	-0.265608
37	C	2.346168	3.086460	-0.064249
38	C	2.432789	1.615939	0.005979
39	C	3.690994	0.982056	0.155659
40	C	3.729474	-0.398739	0.111738
41	H	4.674563	-0.885171	0.296786
42	C	2.611352	-1.170537	-0.243917
43	C	0.782088	0.251553	2.751806
44	H	0.504228	1.293897	2.605995
45	H	1.757366	0.100004	2.286785
46	H	0.907292	0.085800	3.823514
47	C	-4.640255	-1.580325	0.206876
48	H	-5.350582	-1.037141	-0.406618
49	H	-5.129837	-1.855874	1.144833
50	C	-3.795615	3.575975	-0.549283
51	H	-4.497525	3.508002	0.280990
52	H	-4.313975	3.148902	-1.408189
53	H	-3.577259	4.626067	-0.751557
54	C	1.047023	5.044661	-0.720210
55	H	2.070630	5.391273	-0.807512
56	H	0.550757	5.608906	0.072856
57	C	4.985386	1.716303	0.393267
58	H	4.923603	2.375307	1.258627
59	H	5.235899	2.360063	-0.450364
60	H	5.794011	0.999223	0.544427
61	C	3.973578	-3.211202	0.086279
62	H	4.789563	-2.925903	-0.580077
63	H	3.805071	-4.280622	0.020120
64	H	4.238781	-2.948381	1.111797
65	H	0.515914	5.203530	-1.659330
66	H	-4.312183	-2.484565	-0.306091

Total energy: -1663.7098948 (Hartree)

No imaginary frequency.

4. References

[S1] Lee, C.; Yang, W.; Parr, R. G. Development of the Colle-Salvetti correlation-energy formula into a functional of the electron density. *Phys*. *Rev*. *B* **1988**, *37*, 785–789.

[S2] Becke, A. D. Density‐functional thermochemistry. III. The role of exact exchange. *J*. *Chem*. *Phys*. **1993**, *98*, 5648‐5652.

[S3] Krishnan, R.; Binkley, J. S.; Seeger, R.; Pople, J. A. Self-consistent molecular orbital methods. XX. A basis set for correlated wave functions. J. Chem. Phys. **1980**, *72*, 650–654.

[S4] Gaussian 16, Revision C.01, Frisch, M. J.; Trucks, G. W.; Schlegel, H. B.; Scuseria, G. E.; Robb, M. A.; Cheeseman, J. R.; Scalmani, G.; Barone, V.; Petersson, G. A.; Nakatsuji, H.; Li, X.; Caricato, M.; Marenich, A. V.; Bloino, J.; Janesko, B. G.; Gomperts, R.; Mennucci, B.; Hratchian, H. P.; Ortiz, J. V.; Izmaylov, A. F.; Sonnenberg, J. L.; Williams-Young, D.; Ding, F.; Lipparini, F.; Egidi, F.; Goings, J.; Peng, B.; Petrone, A.; Henderson, T.; Ranasinghe, D.; Zakrzewski, V. G.; Gao, J.; Rega, N.; Zheng, G.; Liang, W.; Hada, M.; Ehara, M.; Toyota, K.; Fukuda, R.; Hasegawa, J.; Ishida, M.; Nakajima, T.; Honda, Y.; Kitao, O.; Nakai, H.; Vreven, T.; Throssell, K.; Montgomery, J. A. Jr.; Peralta, J. E.; Ogliaro, F.; Bearpark, M. J.; Heyd, J. J.; Brothers, E. N.; Kudin, K. N.; Staroverov, V. N.; Keith, T. A.; Kobayashi, R.; Normand, J.; Raghavachari, K.; Rendell, A. P.; Burant, J. C.; Iyengar, S. S.; Tomasi, J.; Cossi, M.; Millam, J. M.; Klene, M.; Adamo, C.; Cammi, R.; Ochterski, J. W.; Martin, R. L.; Morokuma, K.; Farkas, O.; Foresman, J. B.; Fox, D. J. Gaussian, Inc., Wallingford CT, 2019.

5. ^{1}H and ^{13}C NMR Spectral Data

^{1}H NMR spectrum of **6** (500 MHz, $CDCl_3$, 22 °C)

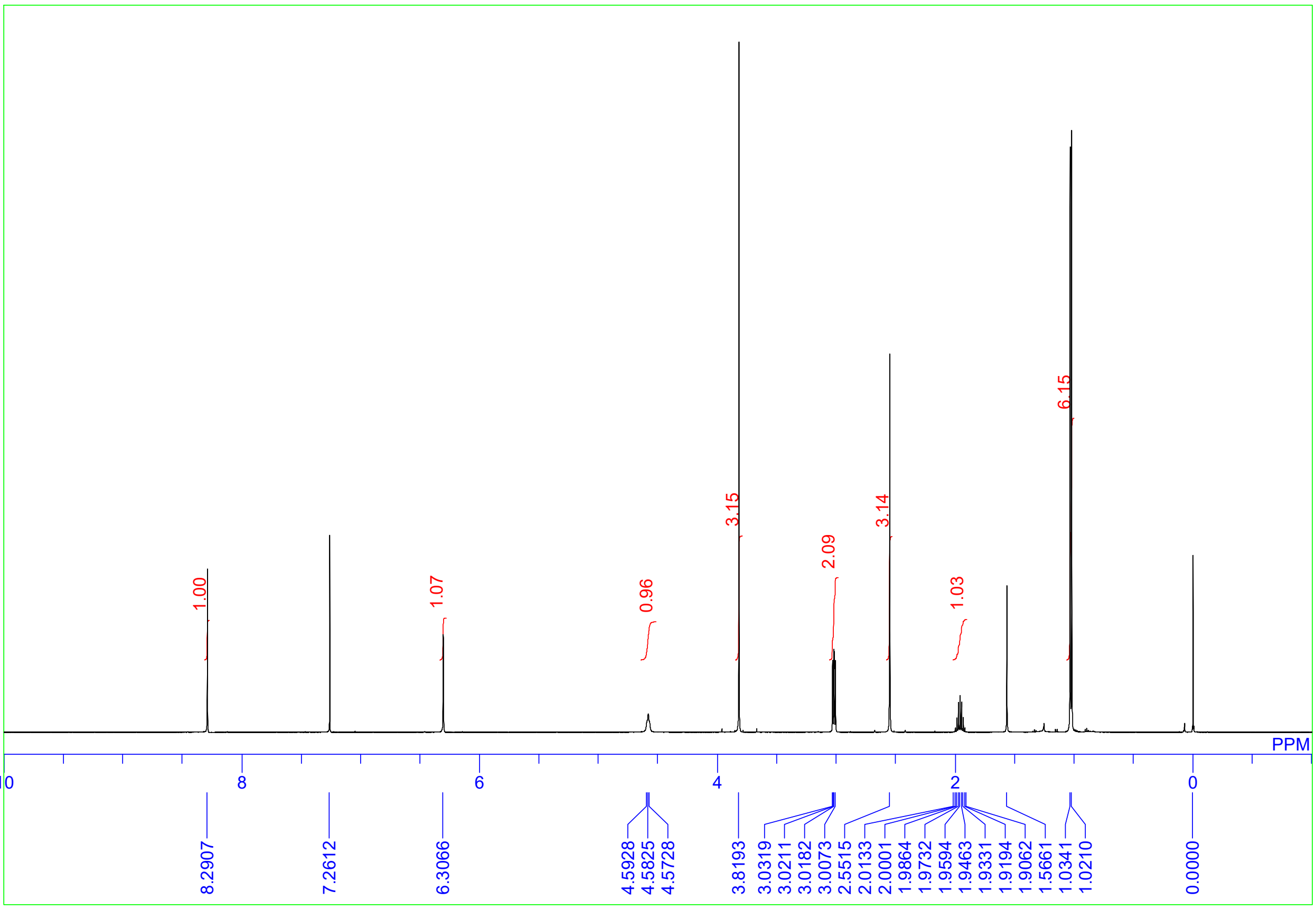

^{13}C NMR spectrum of **6** (126 MHz, $CDCl_3$, 22 °C)

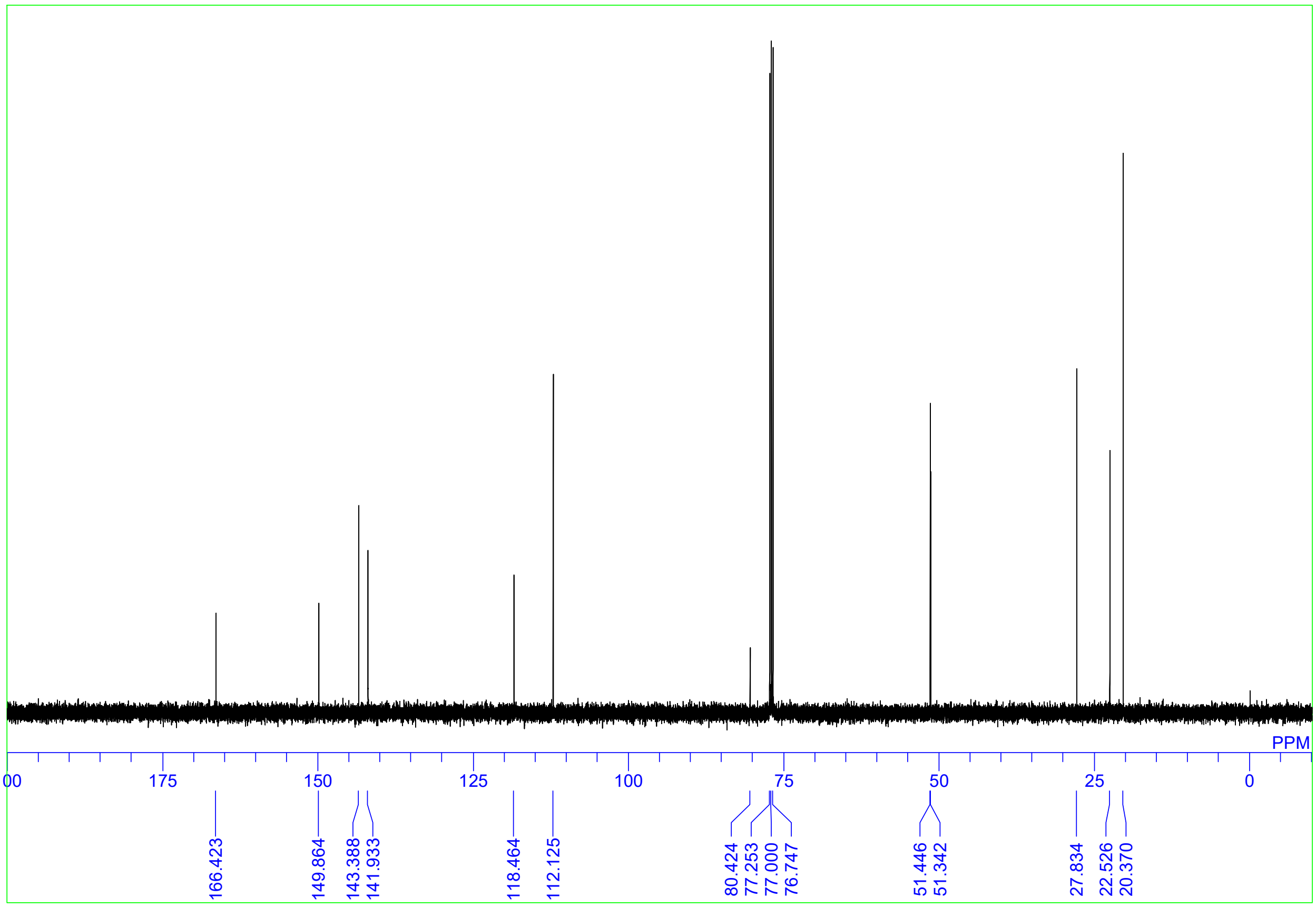

^{1}H NMR spectrum of **7** (500 MHz, $CDCl_3$, 22 °C)

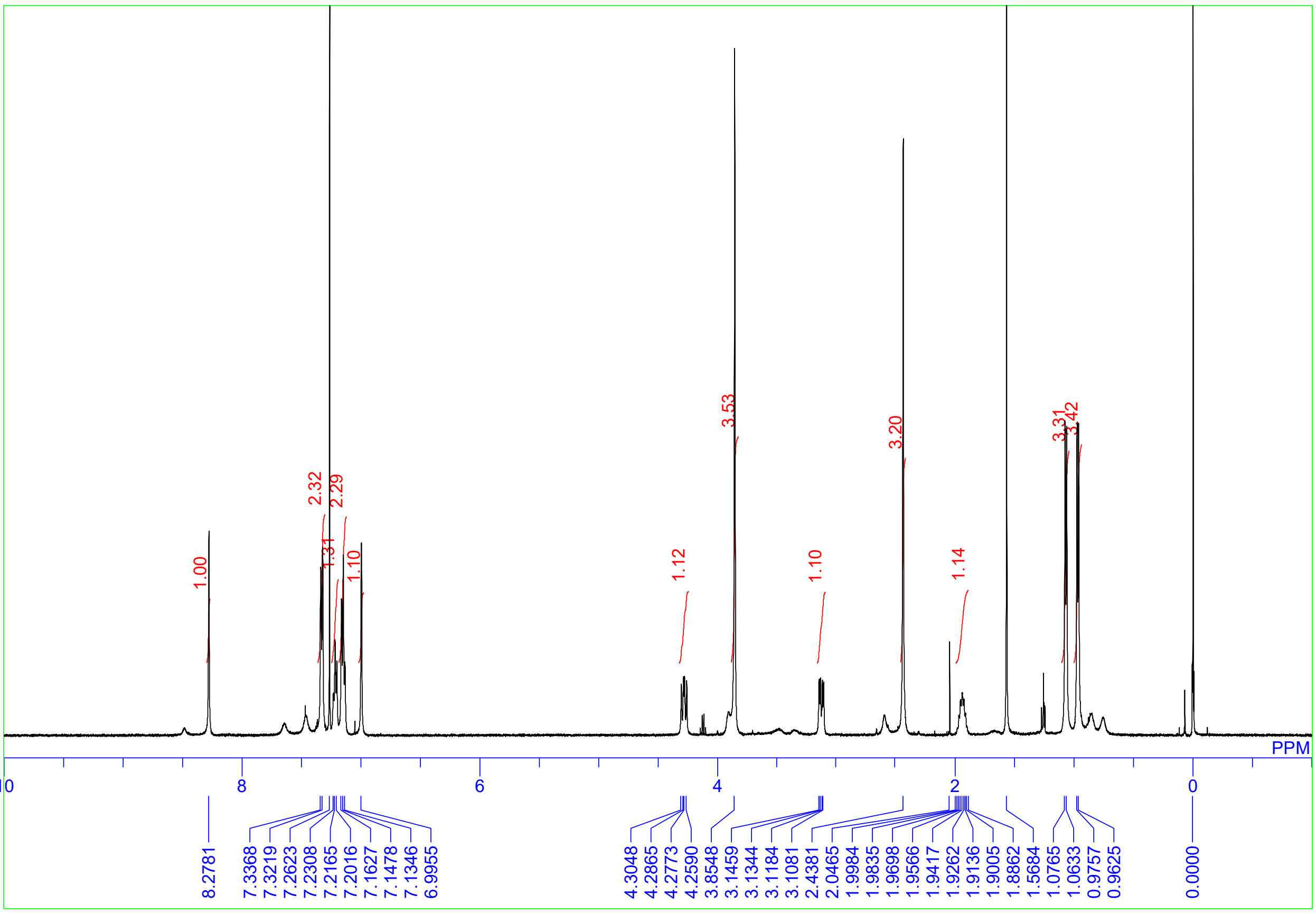

^{13}C NMR spectrum of **7** (126 MHz, $CDCl_3$, 21 °C)

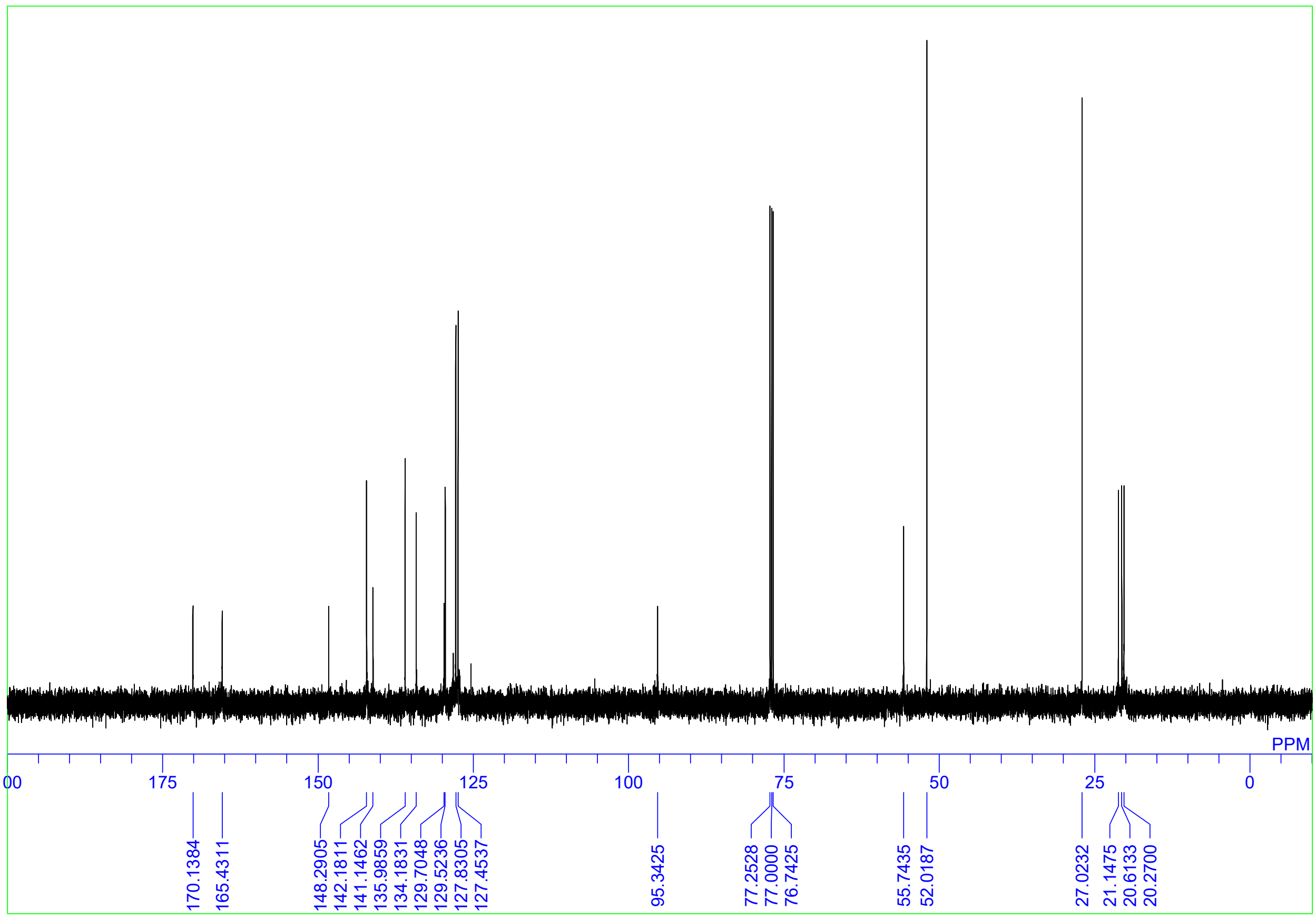

^{1}H NMR spectrum of **8** (500 MHz, $CDCl_3$, 22 °C)

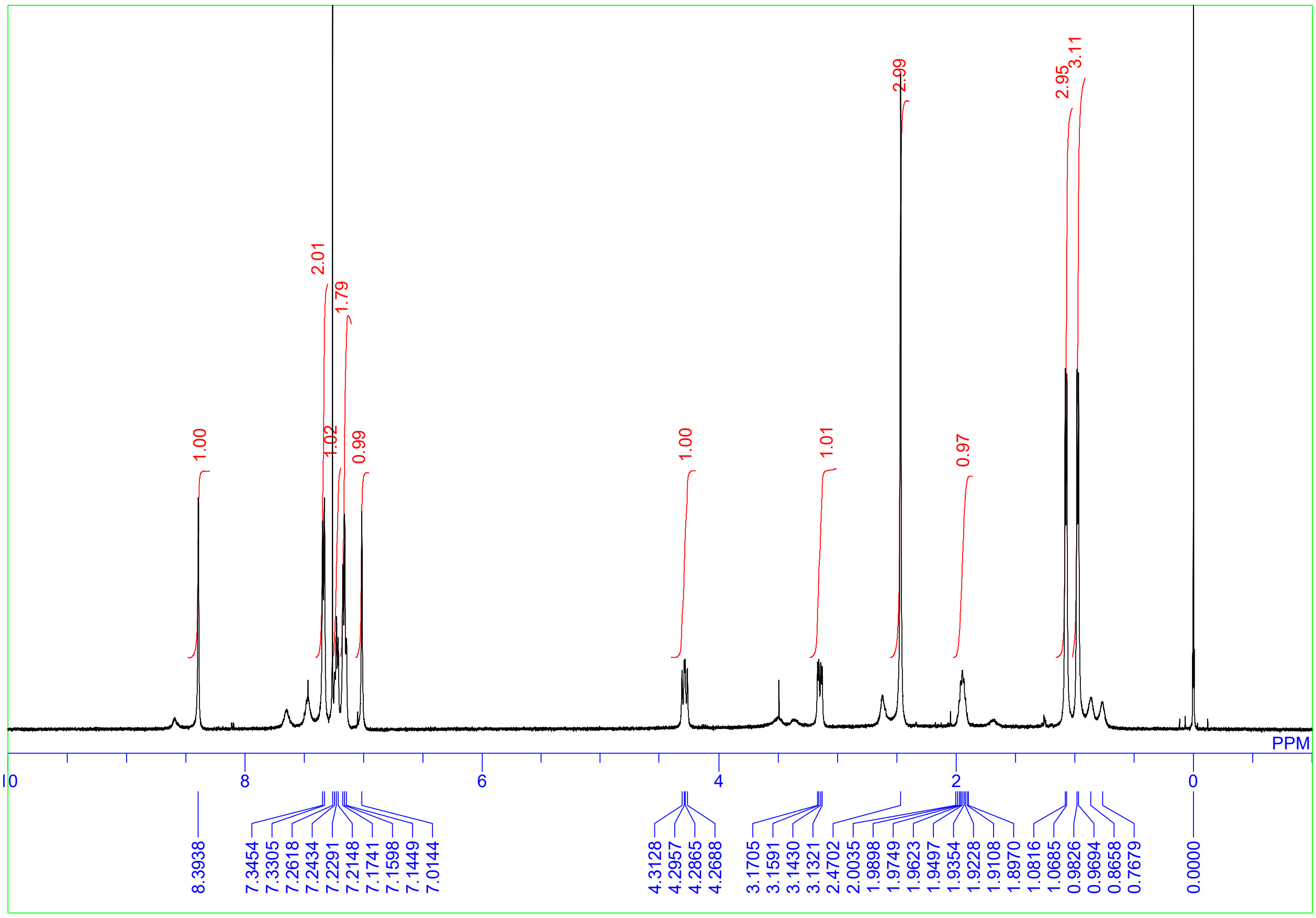

^{13}C NMR spectrum of **8** (126 MHz, $CDCl_3$, 21 °C)

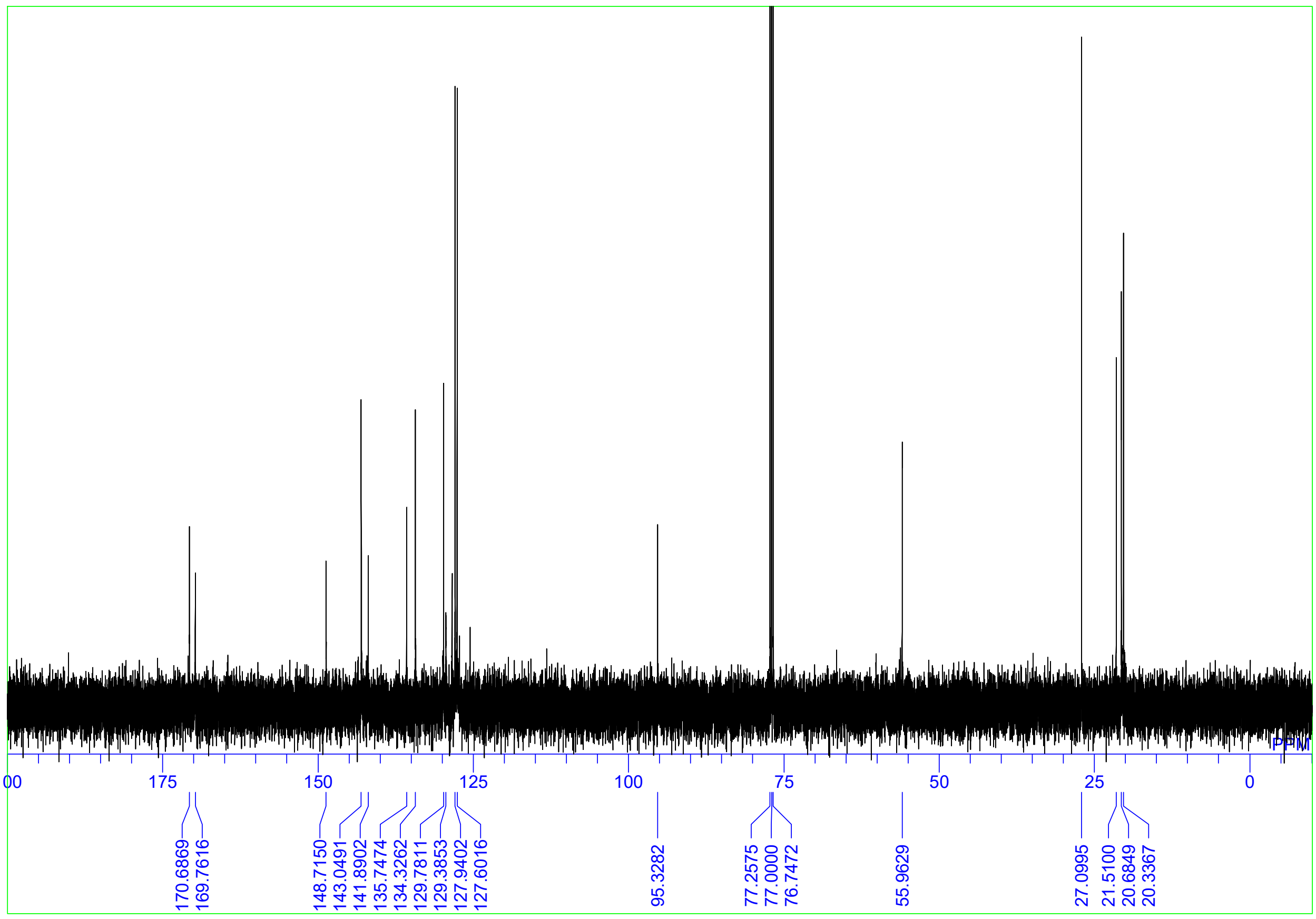

^{1}H NMR spectrum of **9** (500 MHz, $CDCl_3$, 22 °C)

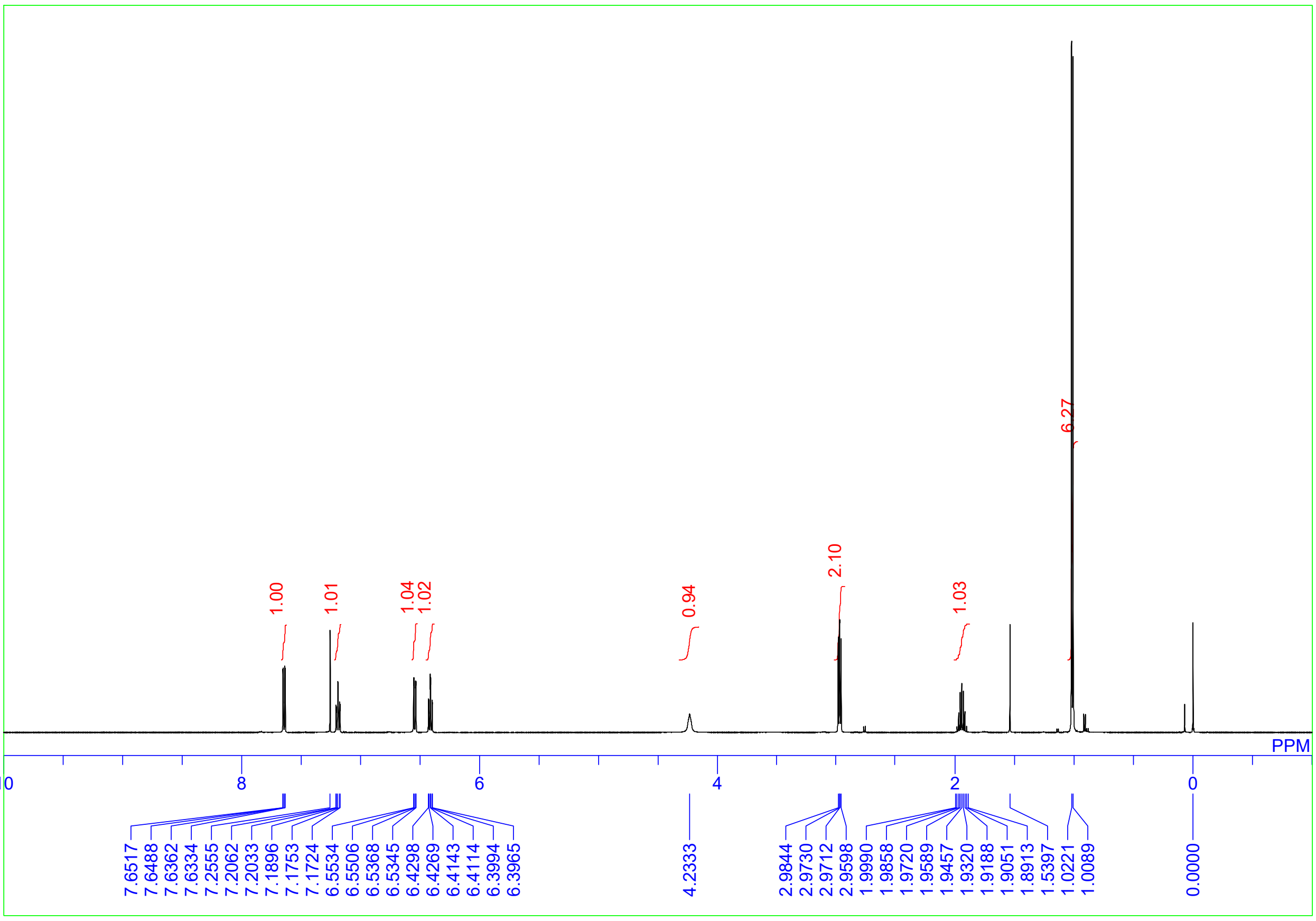

^{13}C NMR spectrum of **9** (126 MHz, $CDCl_3$, 21 °C)

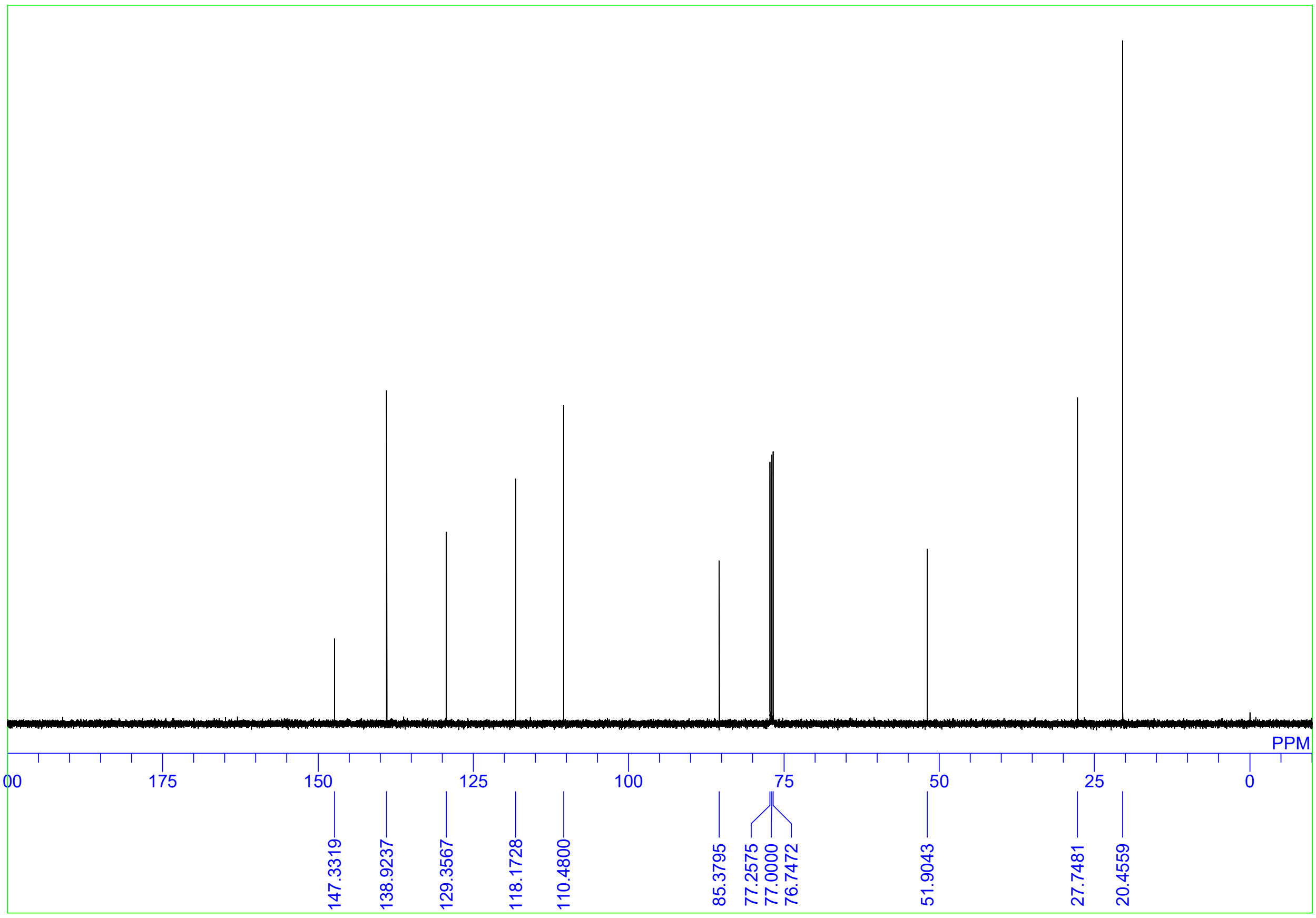

^{1}H NMR spectrum of **3** (500 MHz, DMSO-d_6, 150 °C)

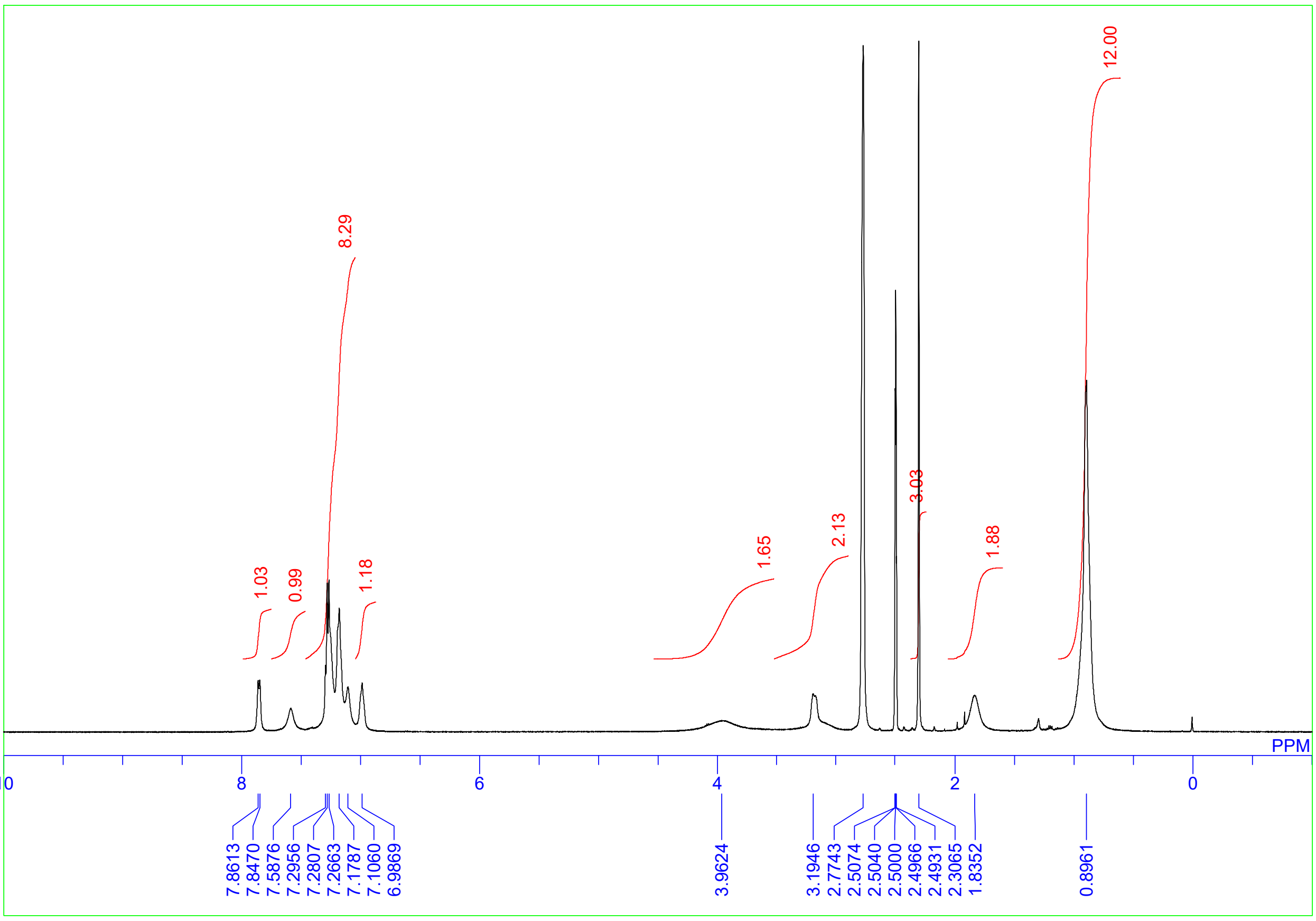

^{13}C NMR spectrum of **3** (126 MHz, DMSO-d_6, 150 °C)

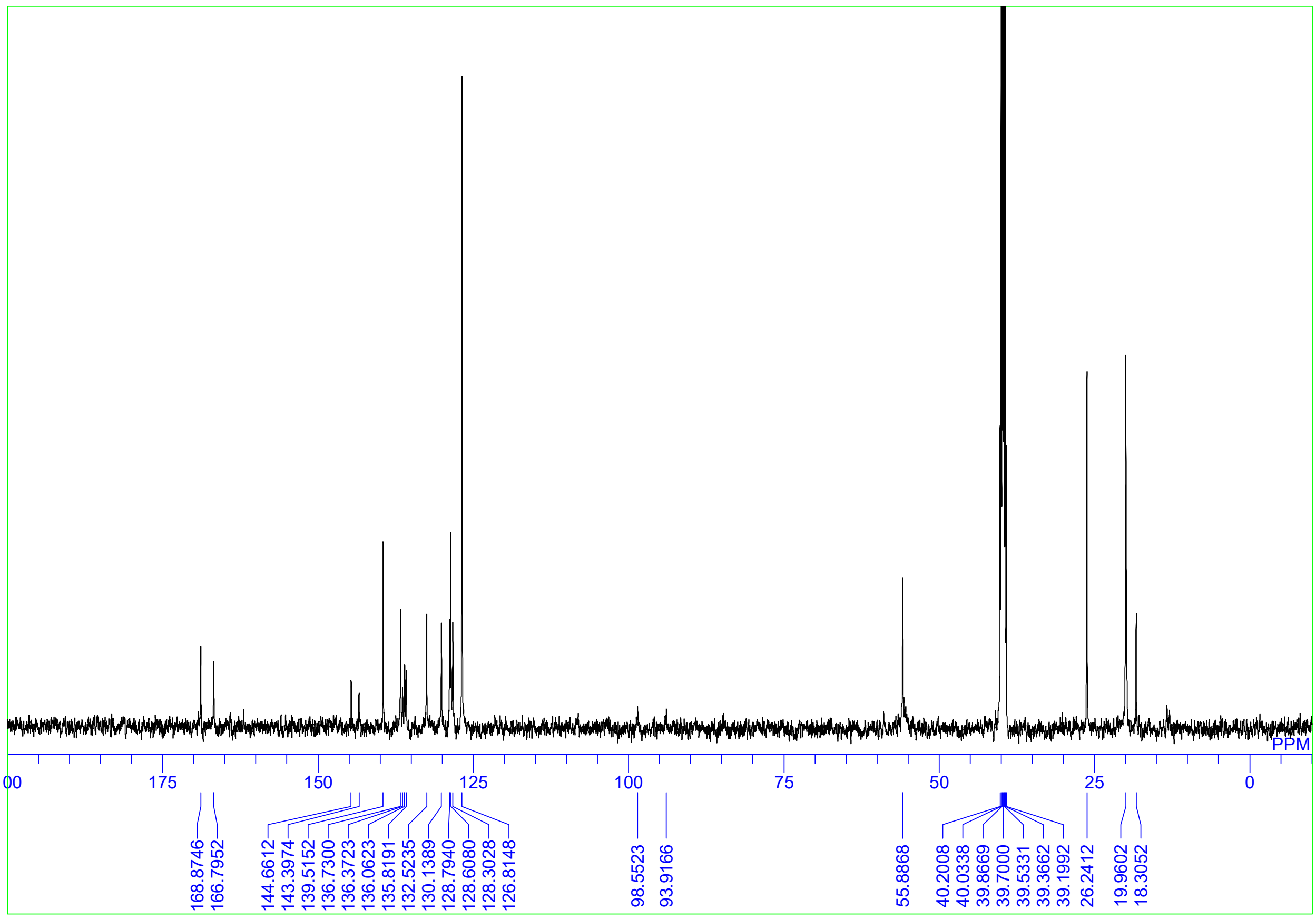

^{1}H NMR spectrum of **1** (500 MHz, $CDCl_3$, 21 °C)

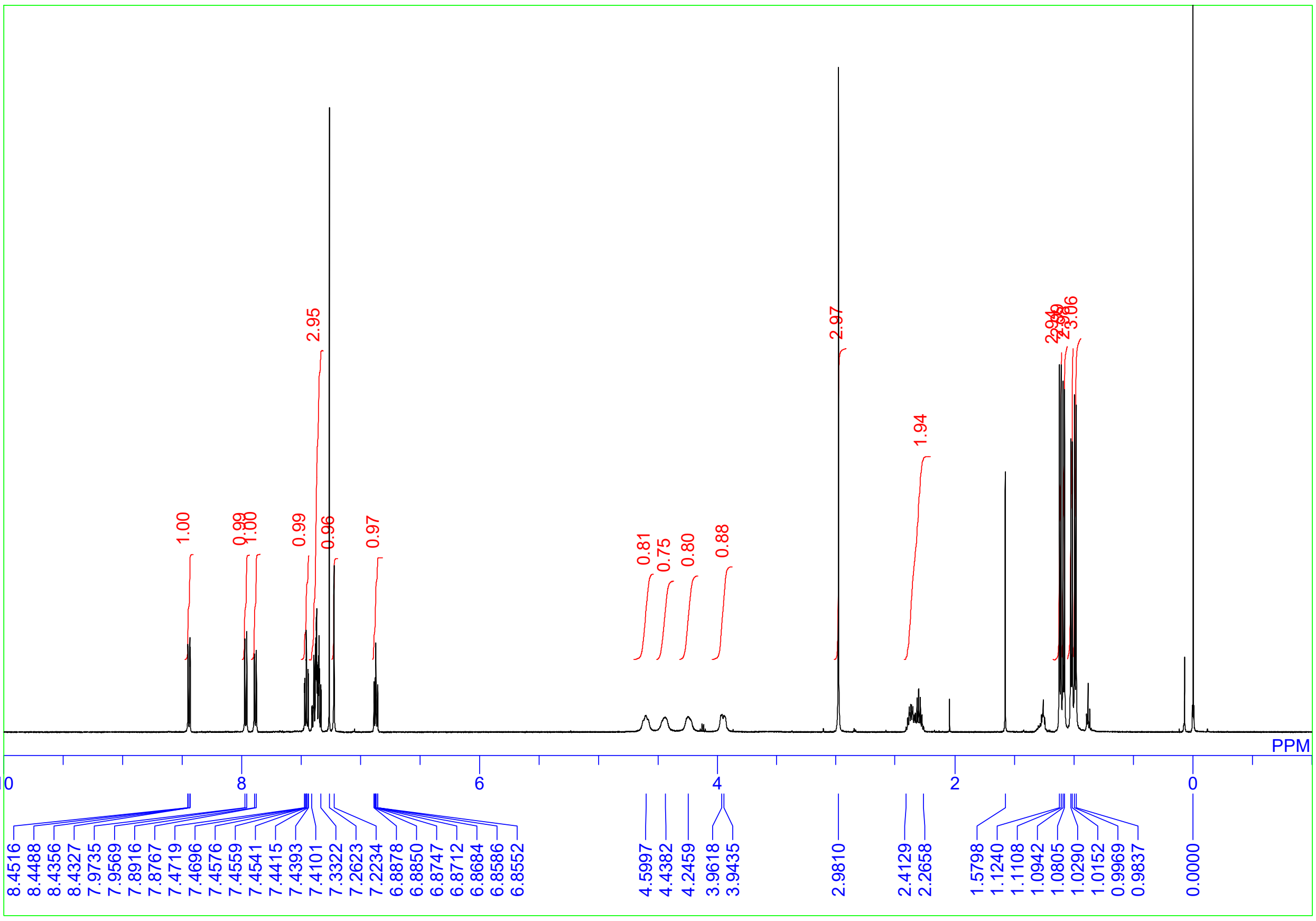

^{13}C NMR spectrum of **1** (126 MHz, $CDCl_3$, 21 °C)

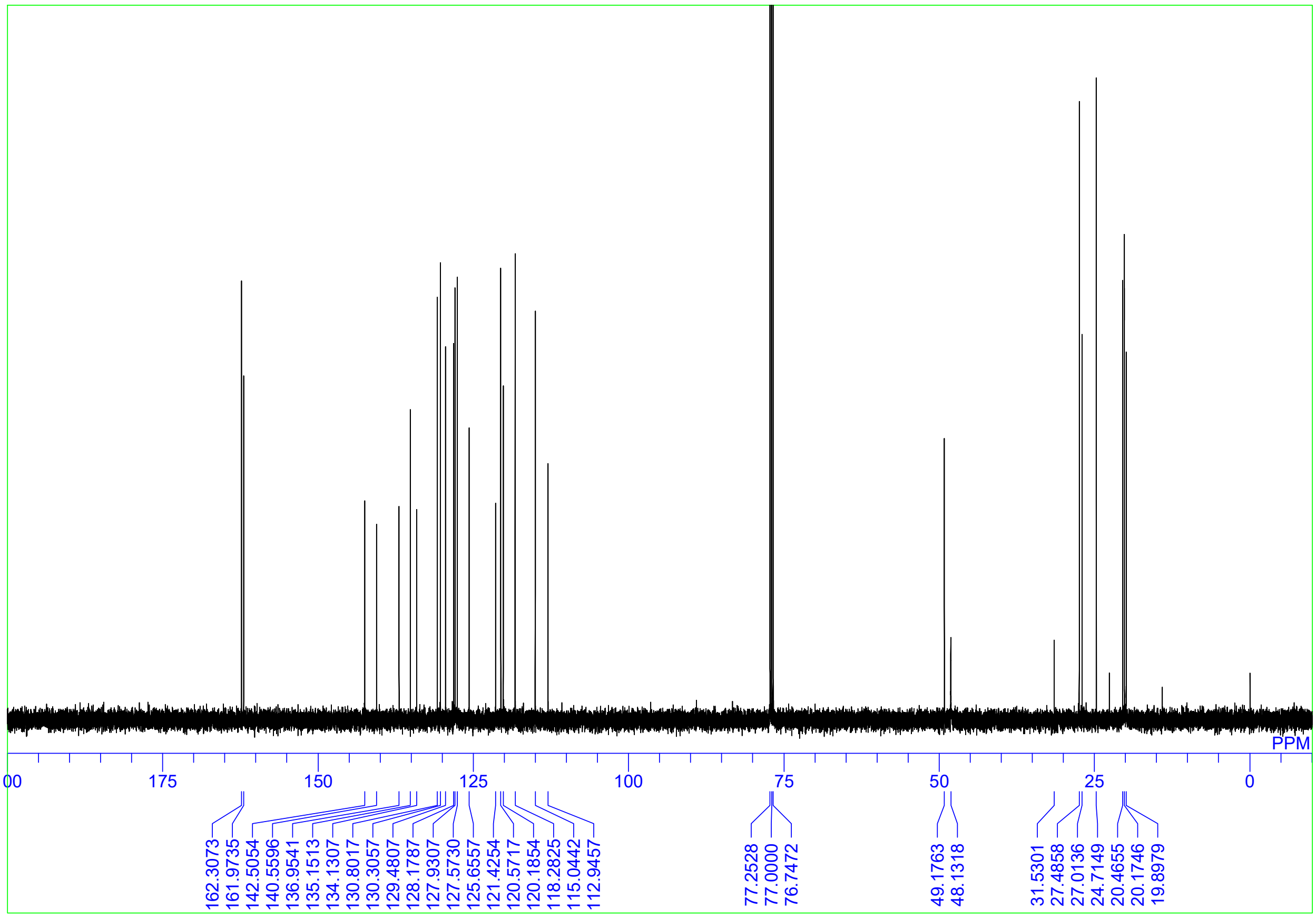

^{1}H NMR spectrum of **10** (500 MHz, $CDCl_3$, 22 °C)

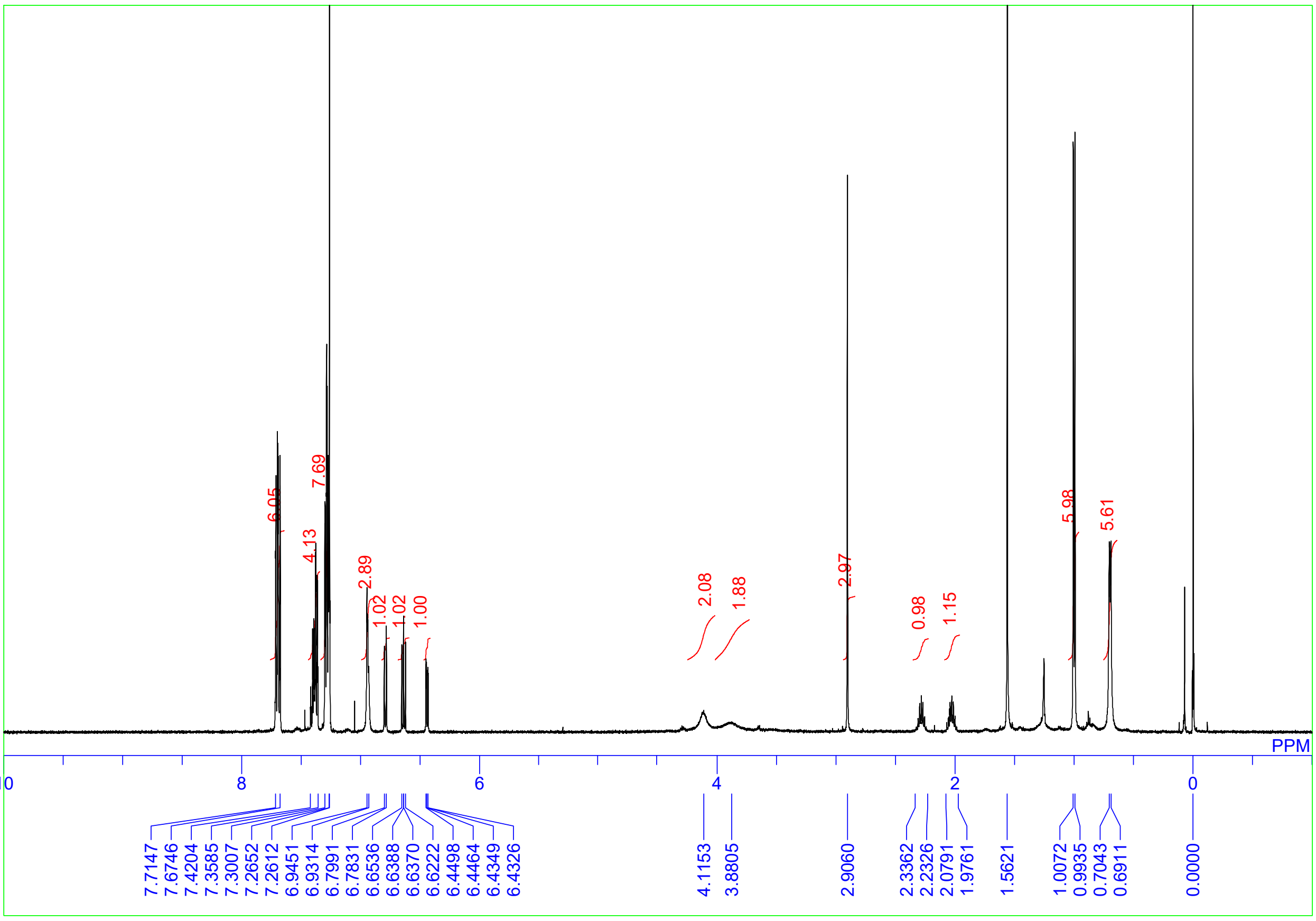

^{13}C NMR spectrum of **10** (126 MHz, $CDCl_3$, 22 °C)

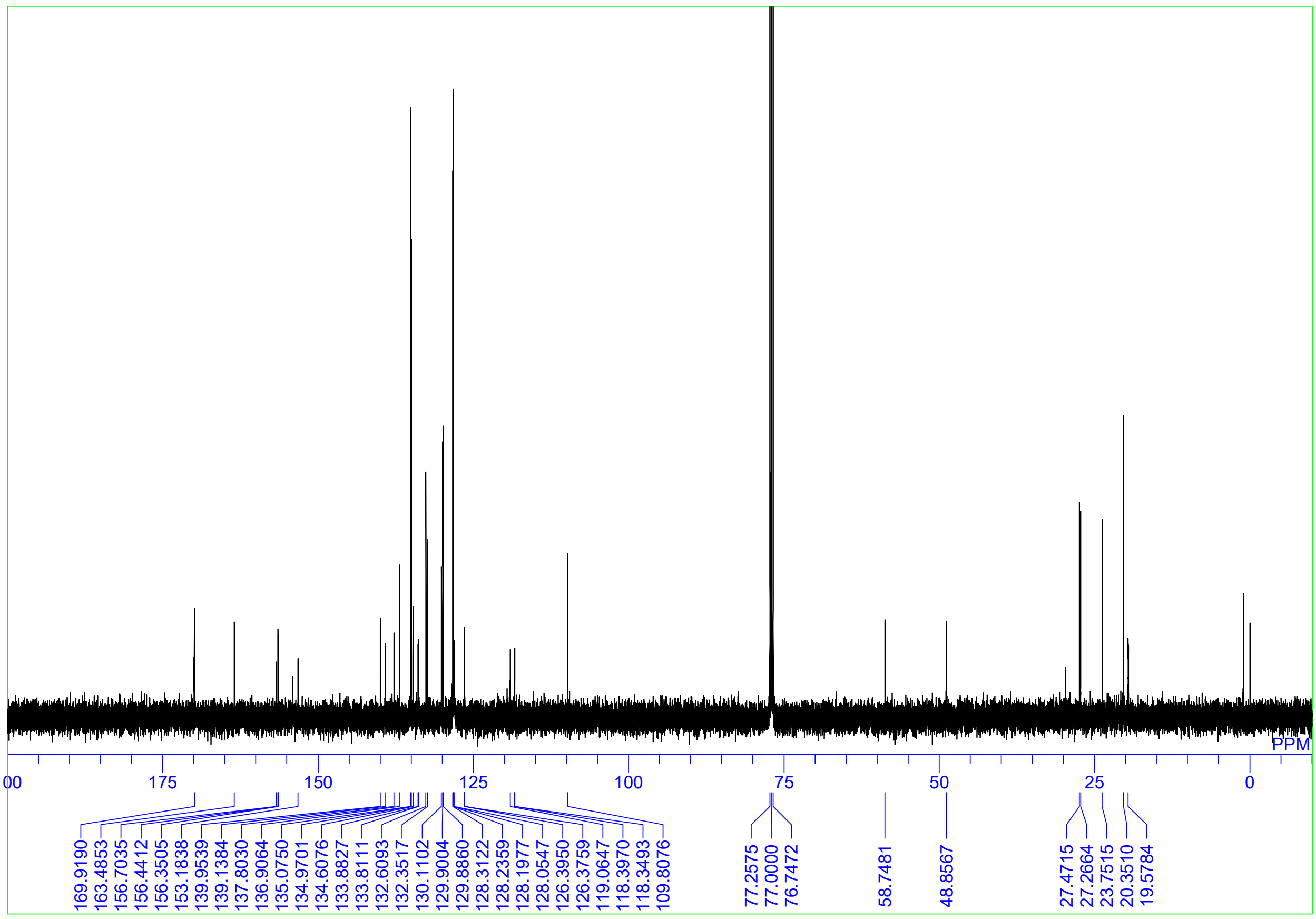

^{1}H NMR spectrum of **11** (500 MHz, DMSO-d_6, 150 °C)

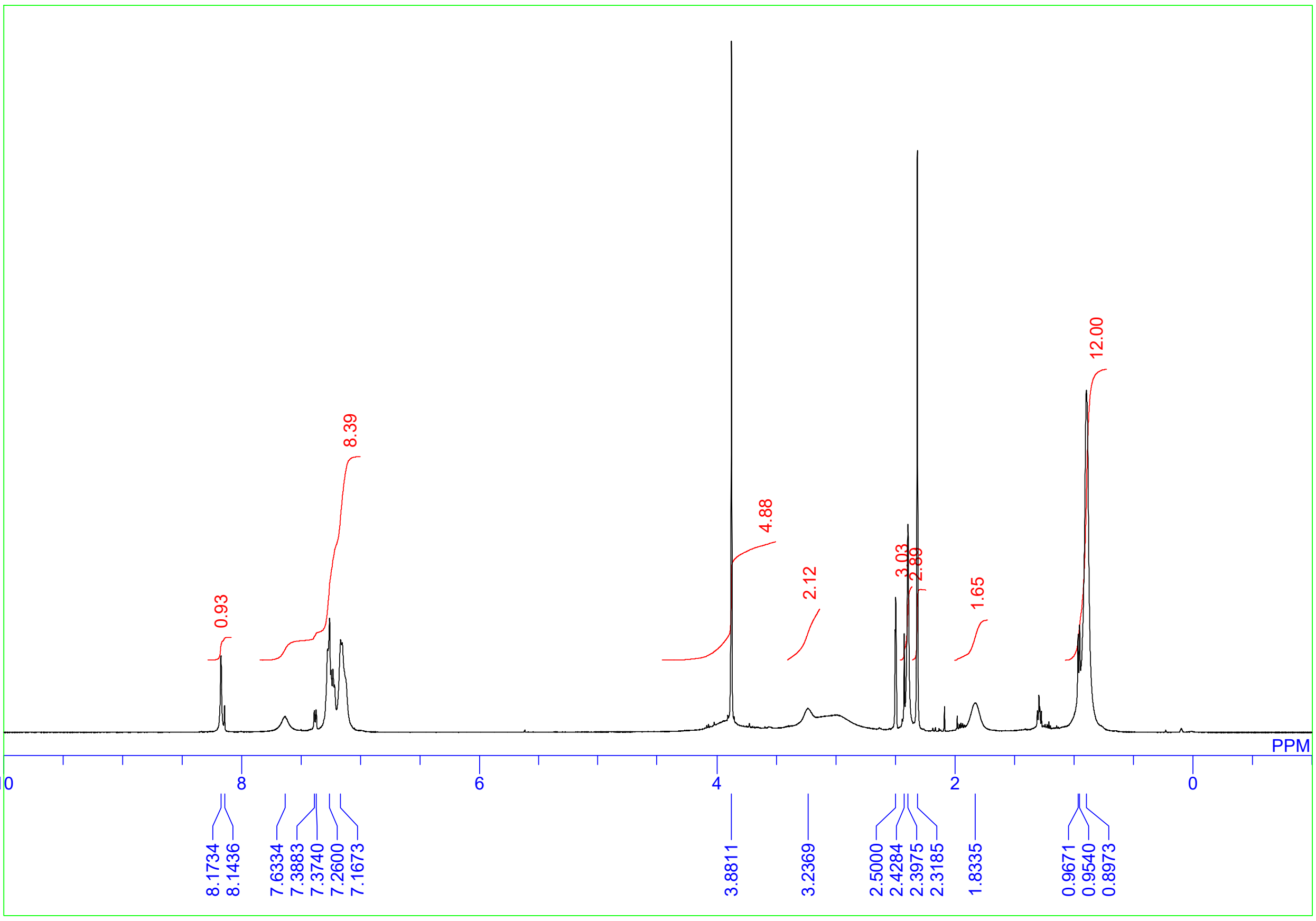

^{13}C NMR spectrum of **11** (126 MHz, DMSO-d_6, 150 °C)

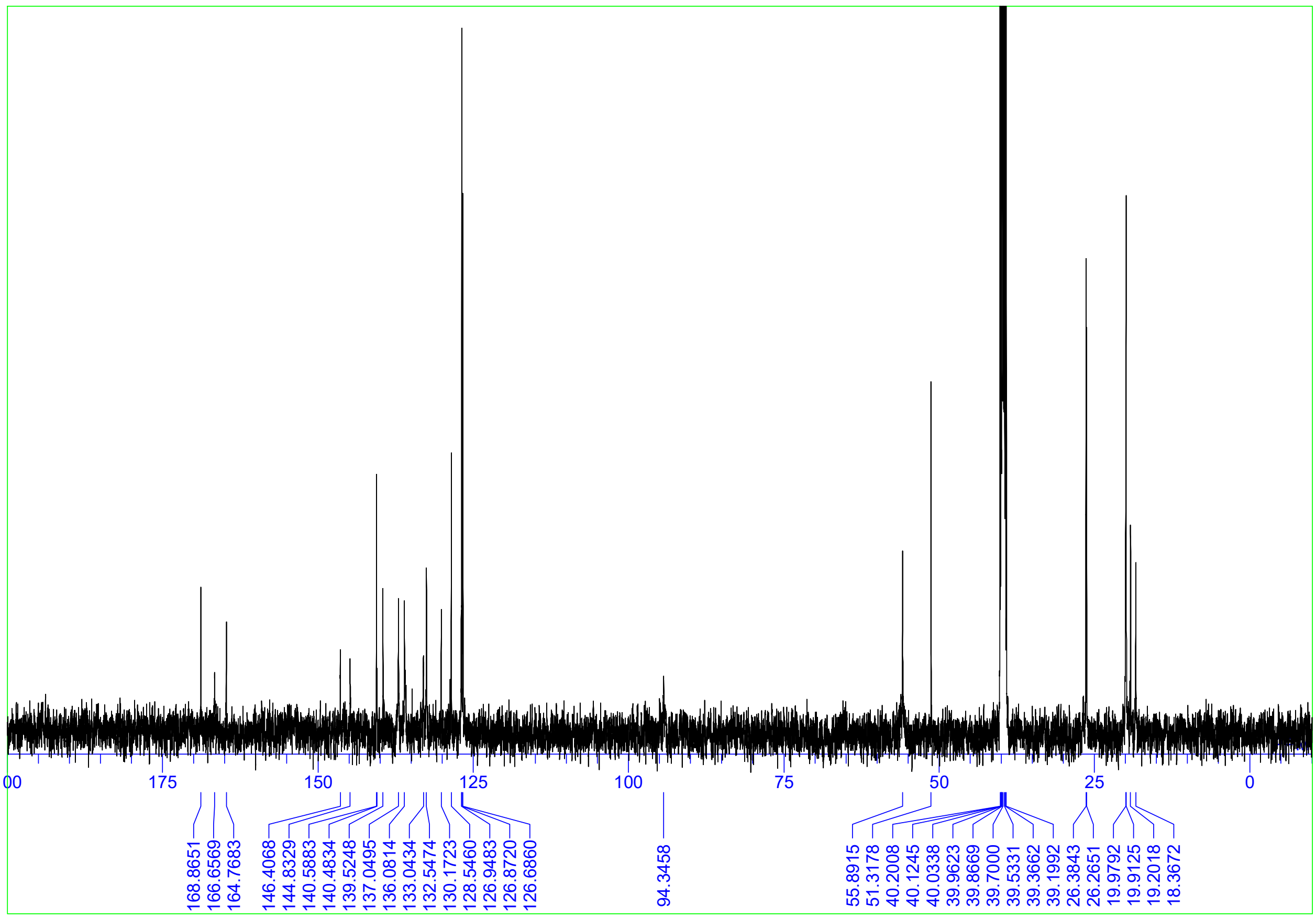

^{1}H NMR spectrum of **12** (500 MHz, DMSO-d_6, 150 °C)

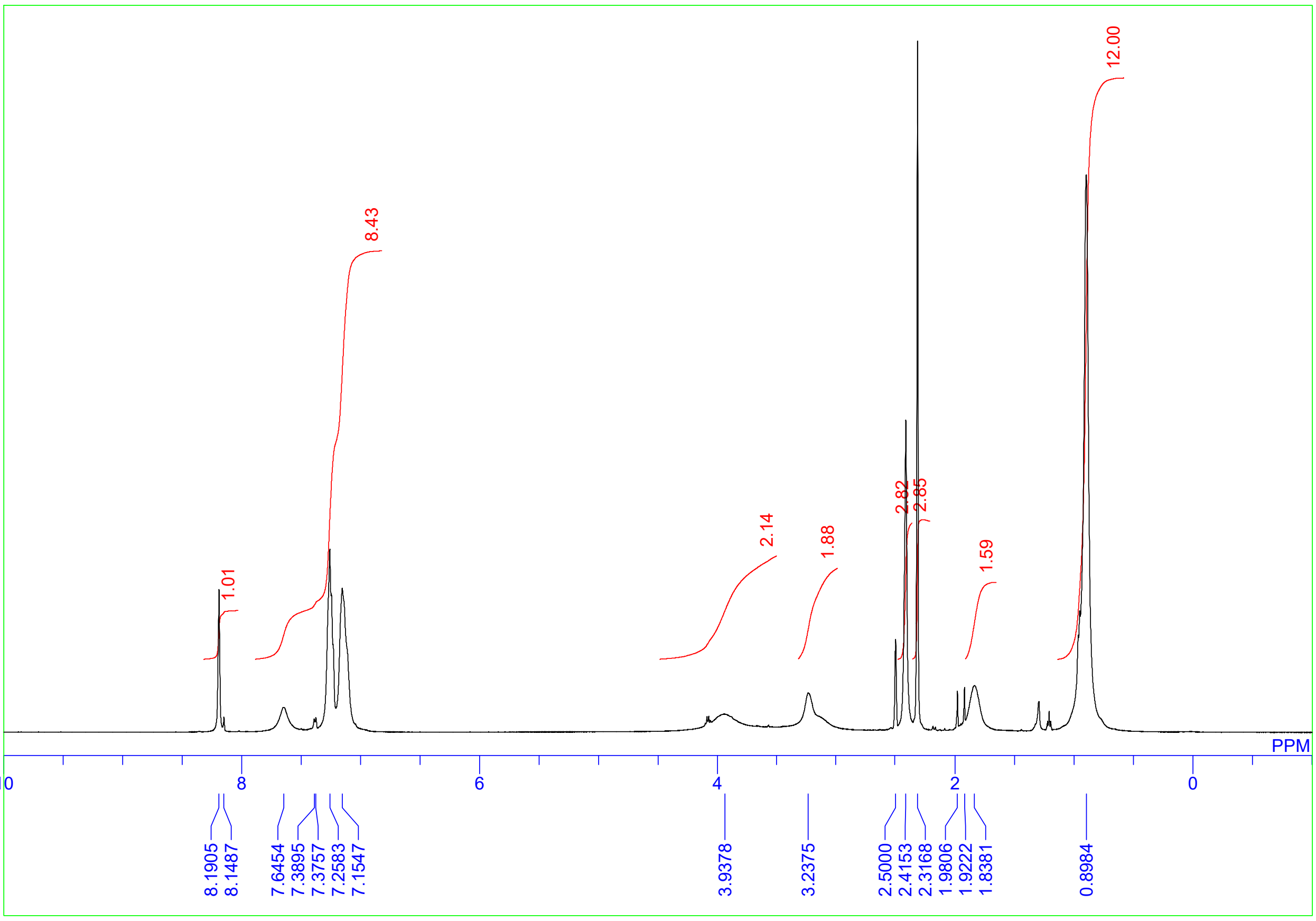

^{13}C NMR spectrum of **12** (126 MHz, DMSO-d_6, 150 °C)

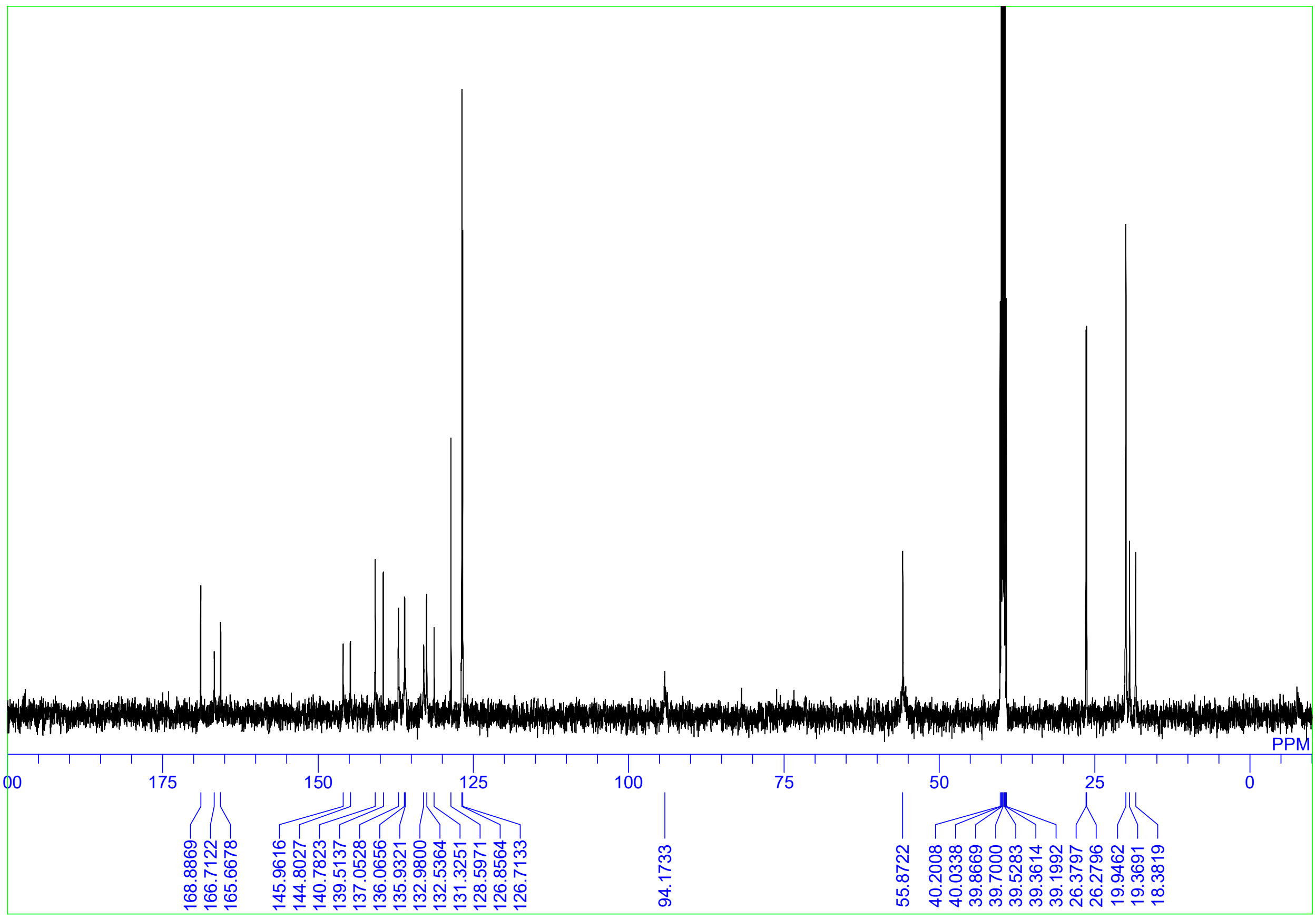

^{1}H NMR spectrum of **4a** (500 MHz, DMSO-d_6, 150 °C)

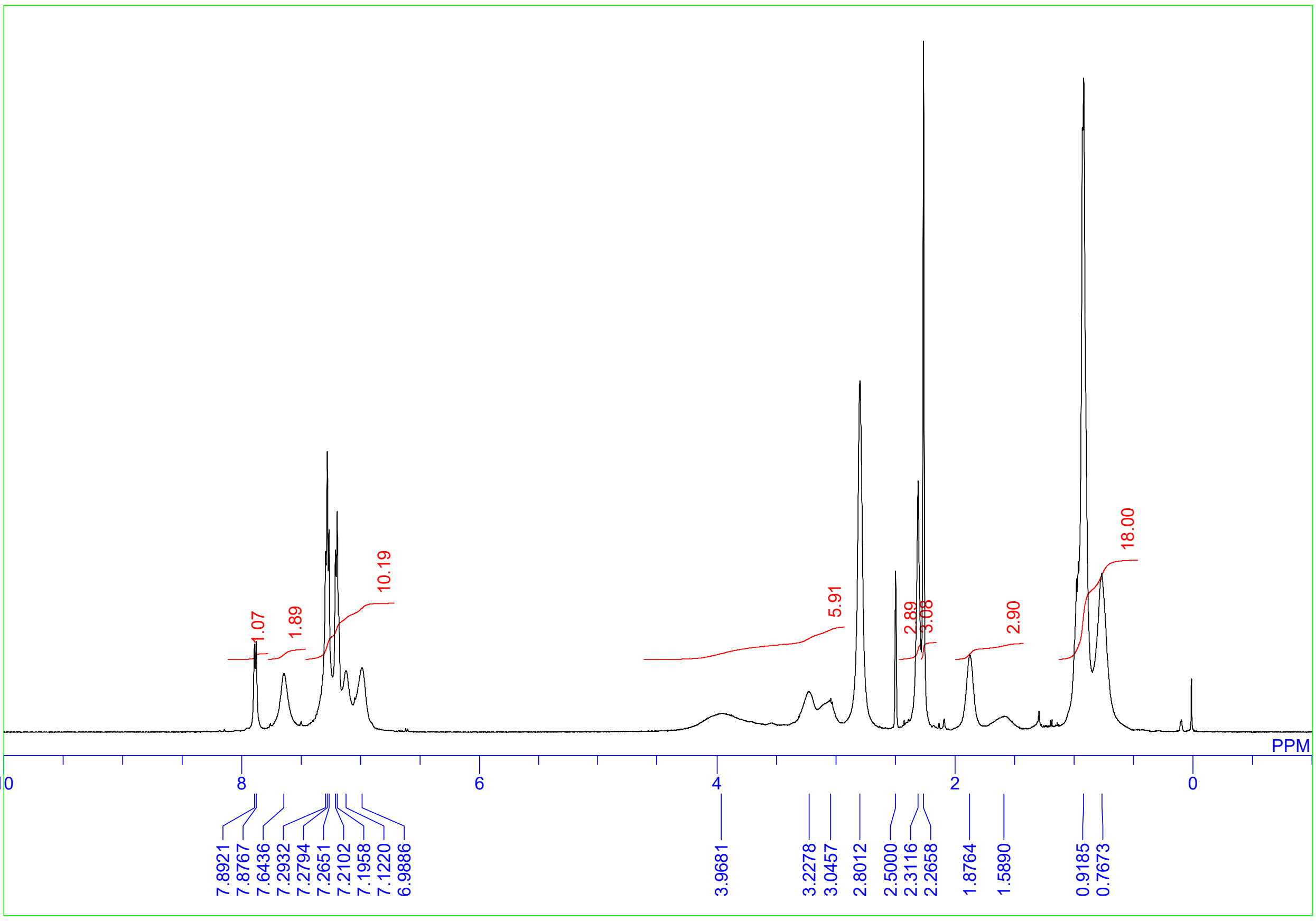

We were unable to obtain an analyzable ^{13}C NMR spectrum (126 MHz, DMSO-d_6, 150 °C) of **4a** probably due to the slow interconversion between the conformers even at 150 °C indicated by broad signals of ^{1}H NMR spectrum at 150 °C.

^{1}H NMR spectrum of **13** (500 MHz, $CDCl_3$, 21 °C)

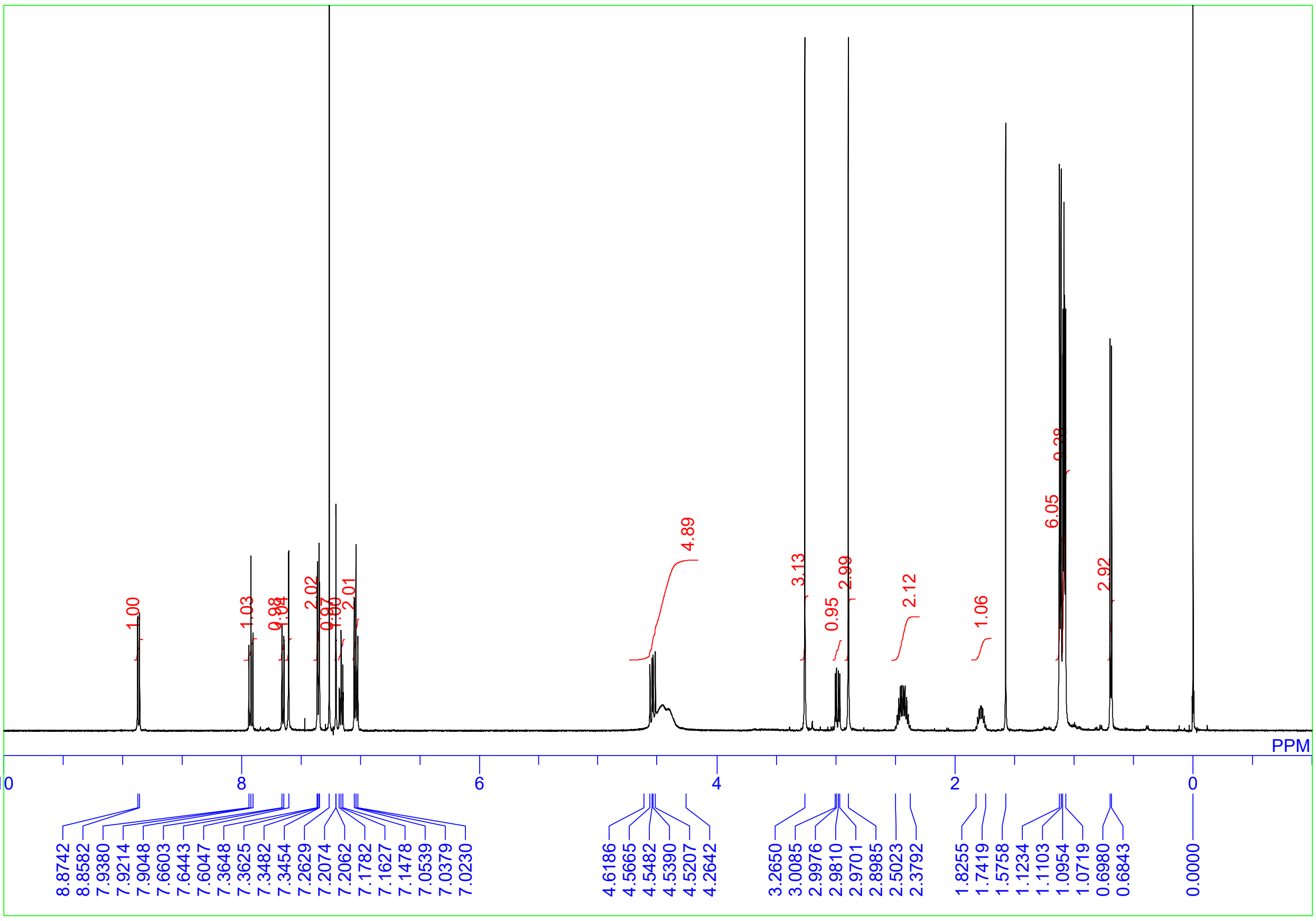

^{13}C NMR spectrum of **13** (126 MHz, $CDCl_3$, 22 °C)

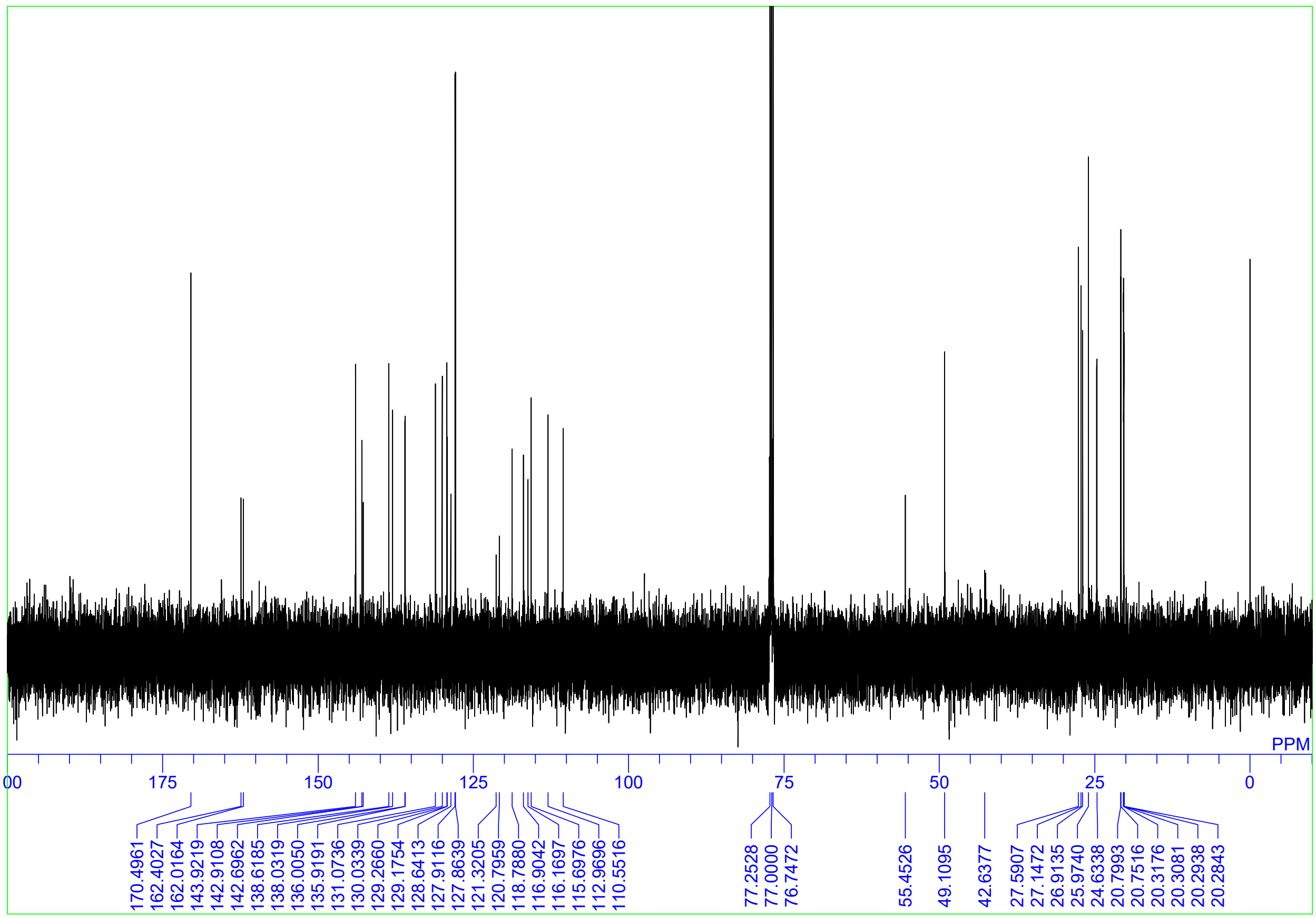

^{1}H NMR spectrum of **14** (500 MHz, $CDCl_3$, 22 °C)

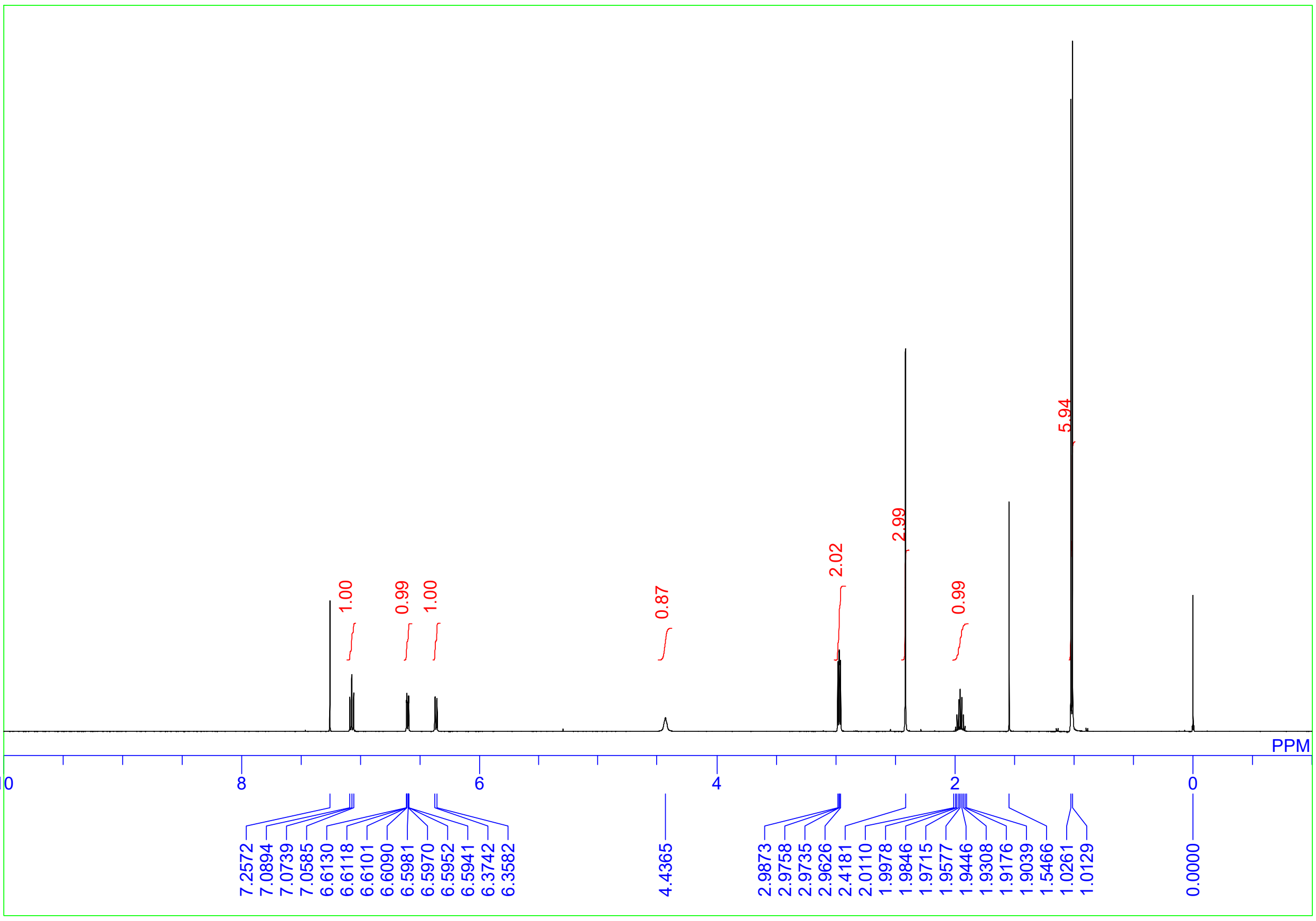

^{13}C NMR spectrum of **14** (126 MHz, $CDCl_3$, 22 °C)

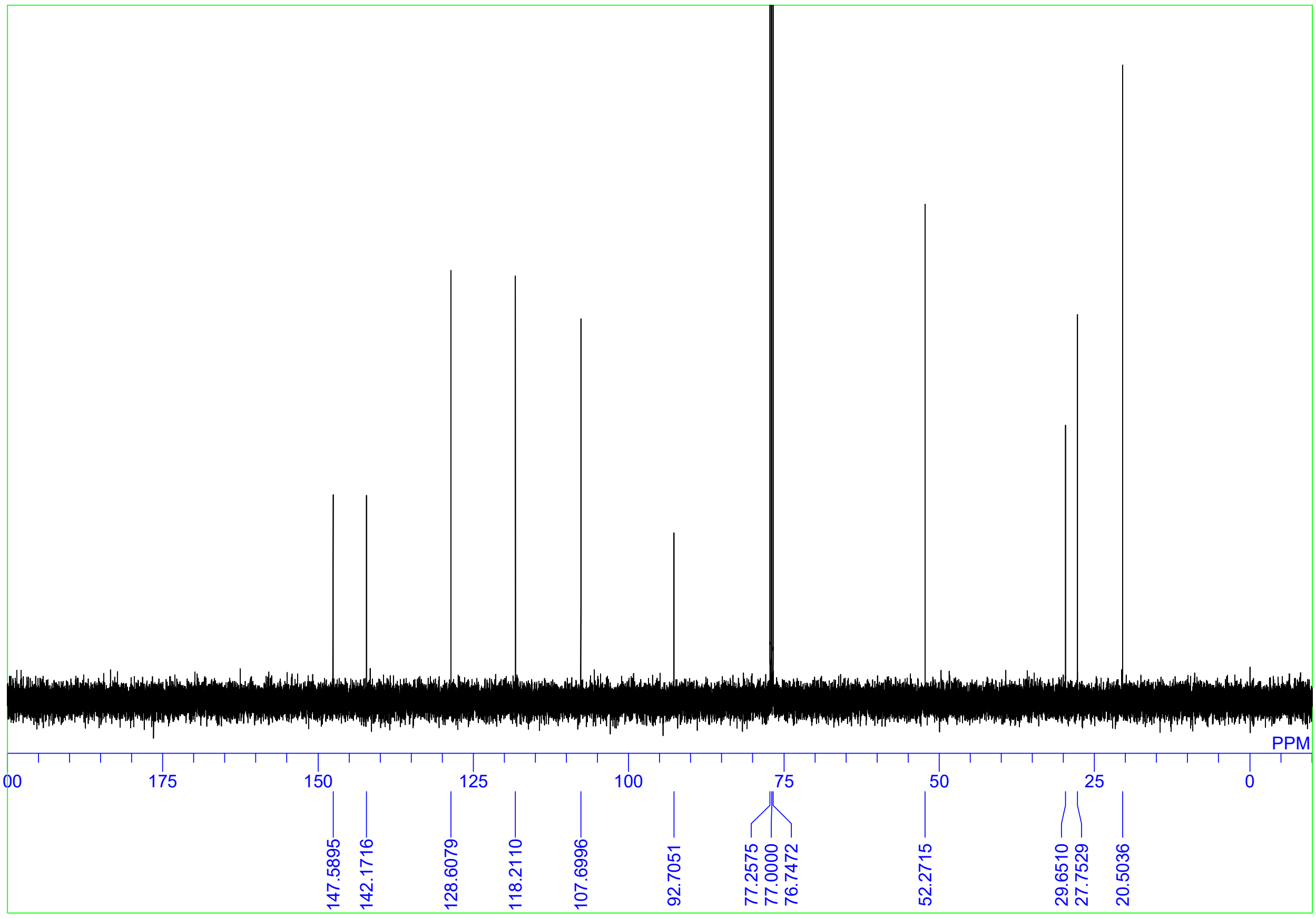

^{1}H NMR spectrum of **4b** (500 MHz, DMSO-d_6, 150 °C)

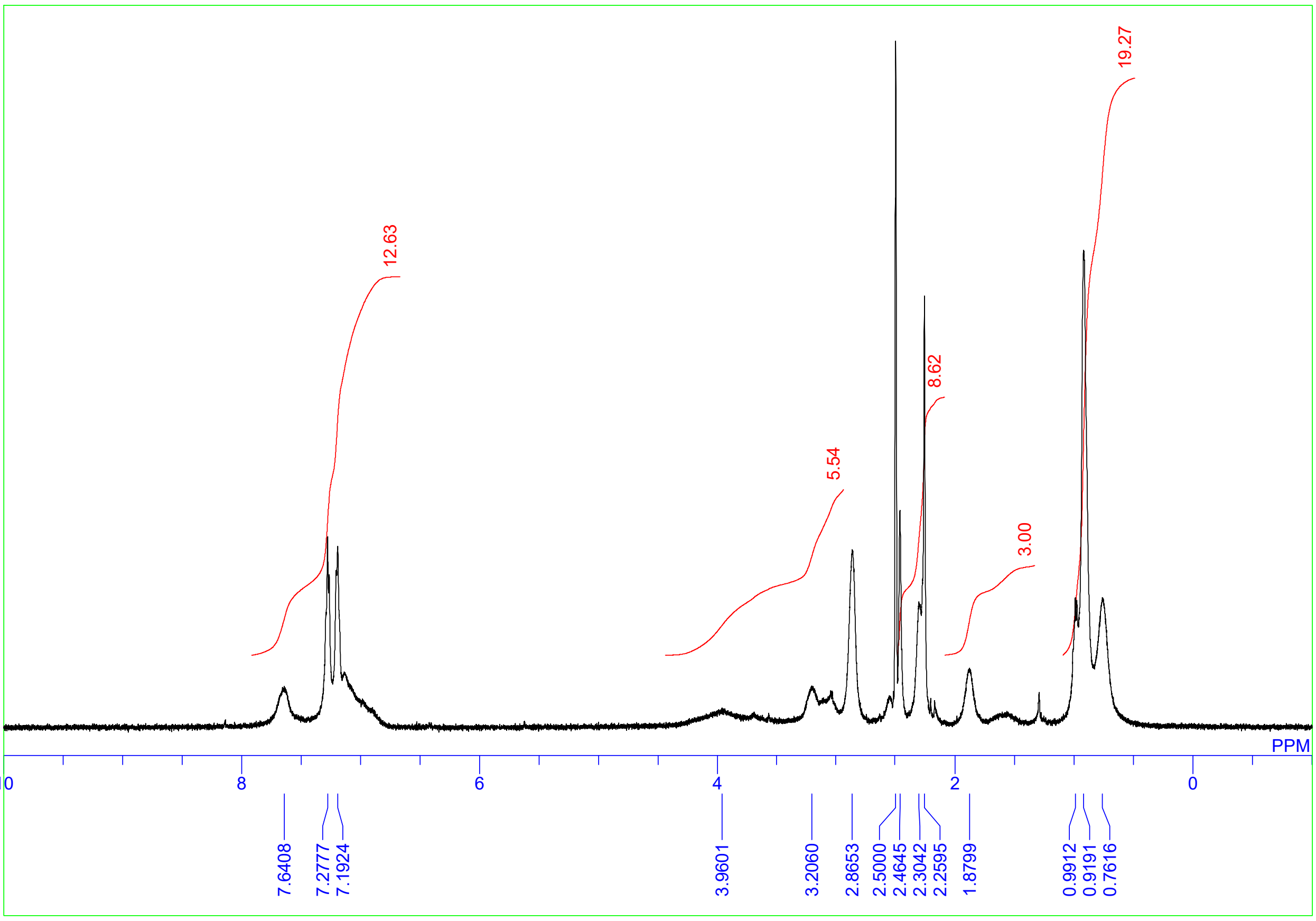

We were unable to obtain an analyzable ^{13}C NMR spectrum (126 MHz, DMSO-d_6, 150 °C) of **4b** probably due to the slow interconversion between the conformers even at 150 °C indicated by broad signals of ^{1}H NMR spectrum at 150 °C.

^{1}H NMR spectrum of **2b** (500 MHz, $CDCl_3$, 22 °C)

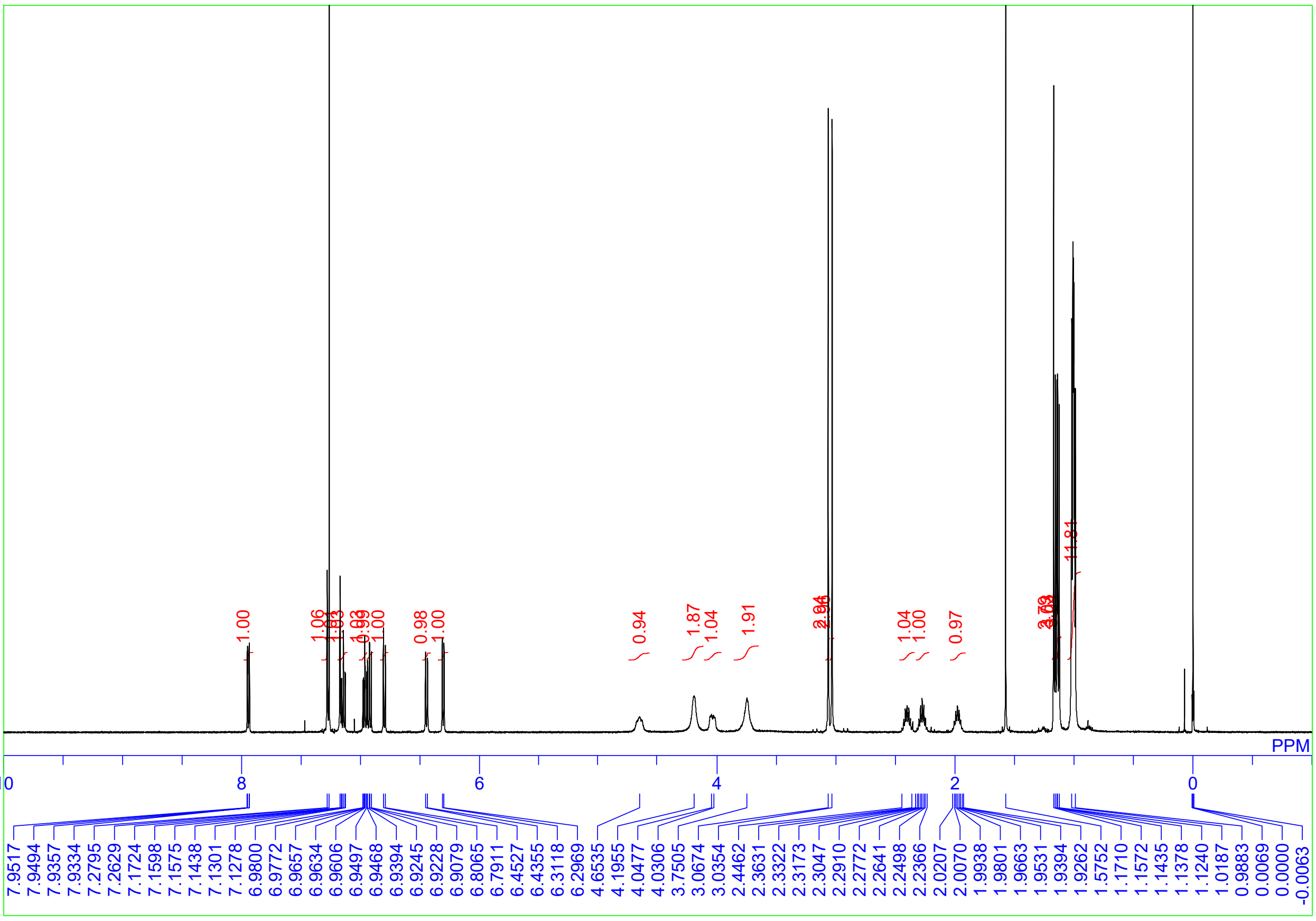

^{13}C NMR spectrum of **2b** (126 MHz, $CDCl_3$, 22 °C)

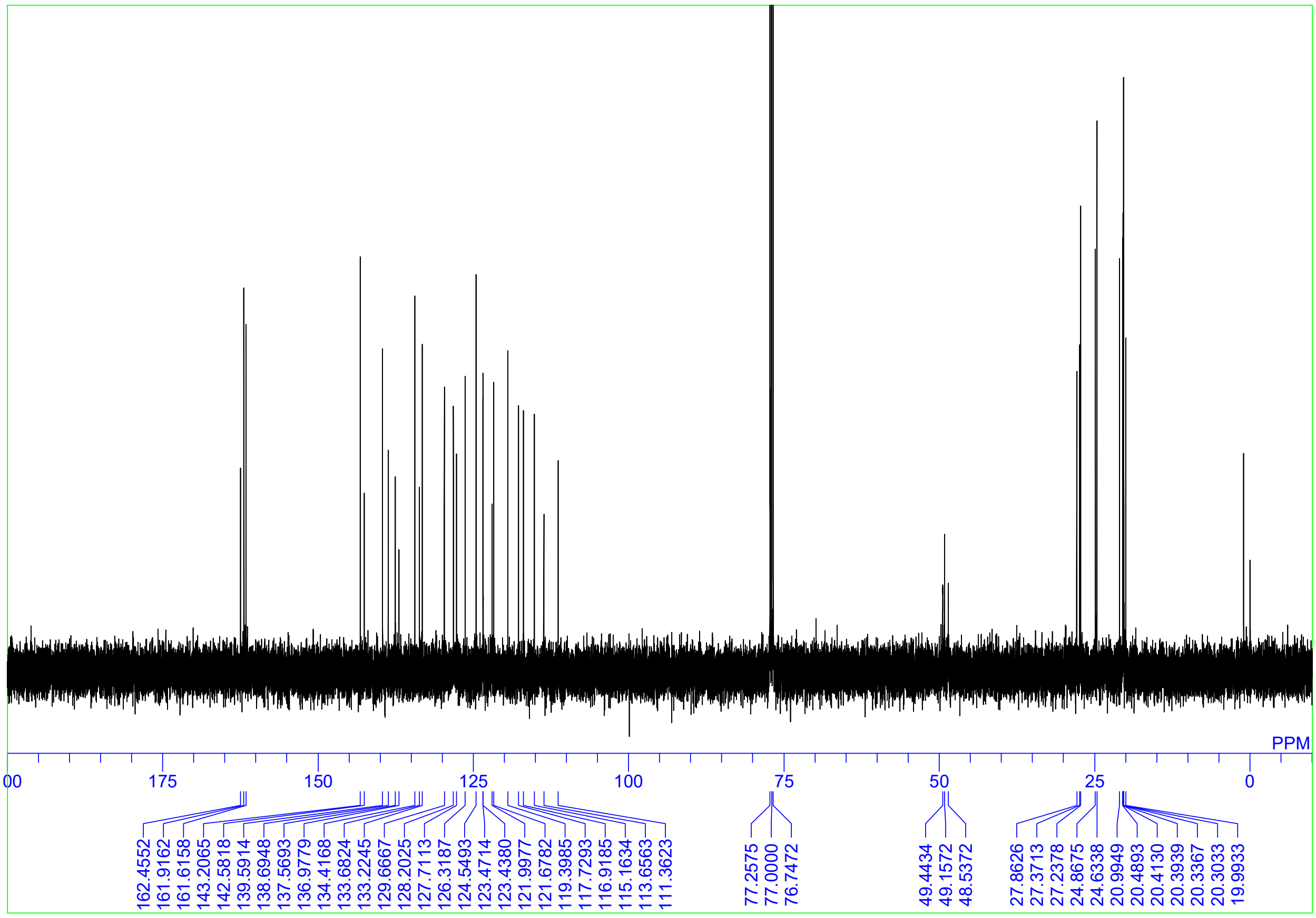

^{1}H NMR spectrum of **15** (500 MHz, DMSO-d_6, 120 °C)

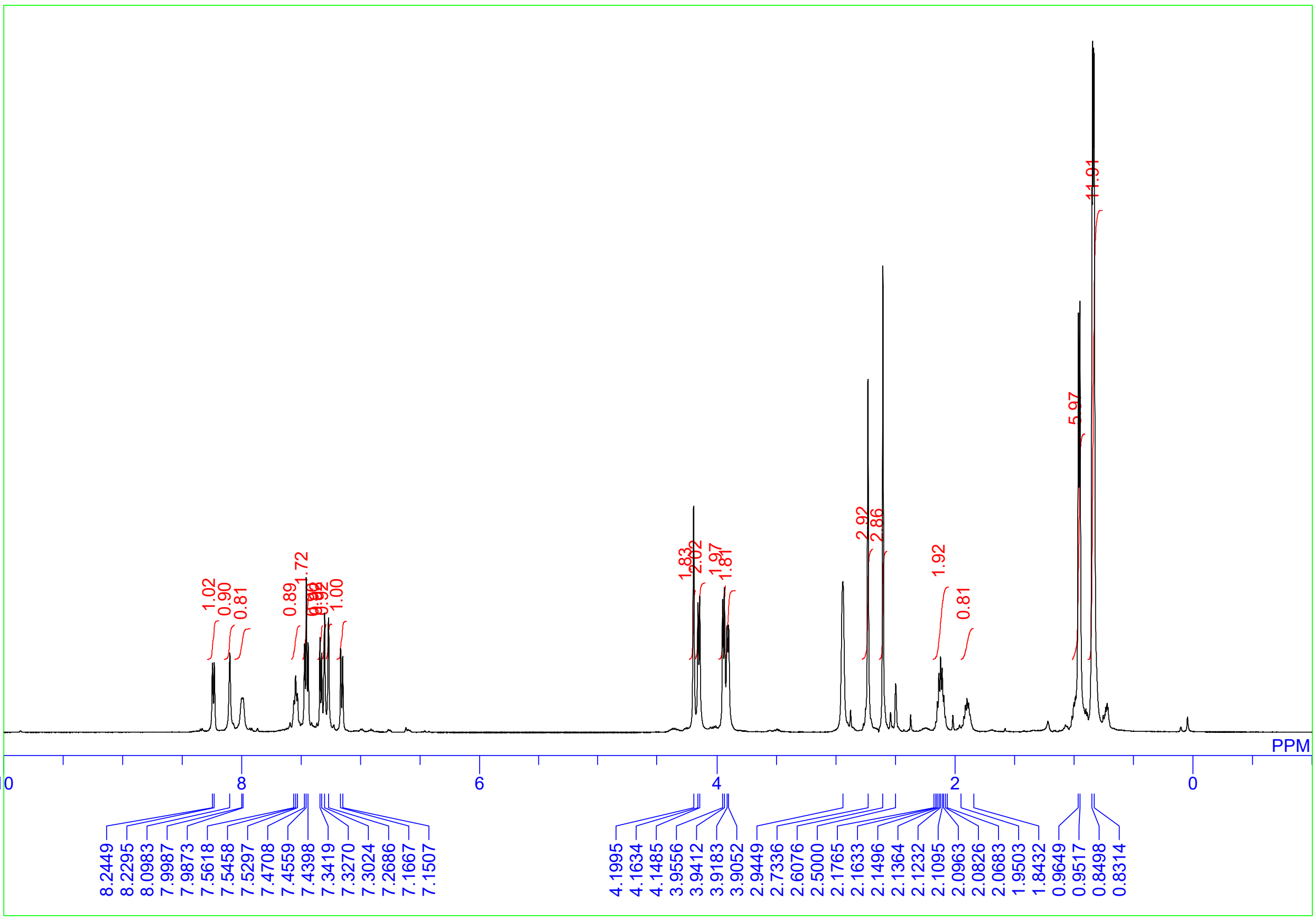

^{13}C NMR spectrum of **15** (126 MHz, DMSO-d_6, 120 °C)

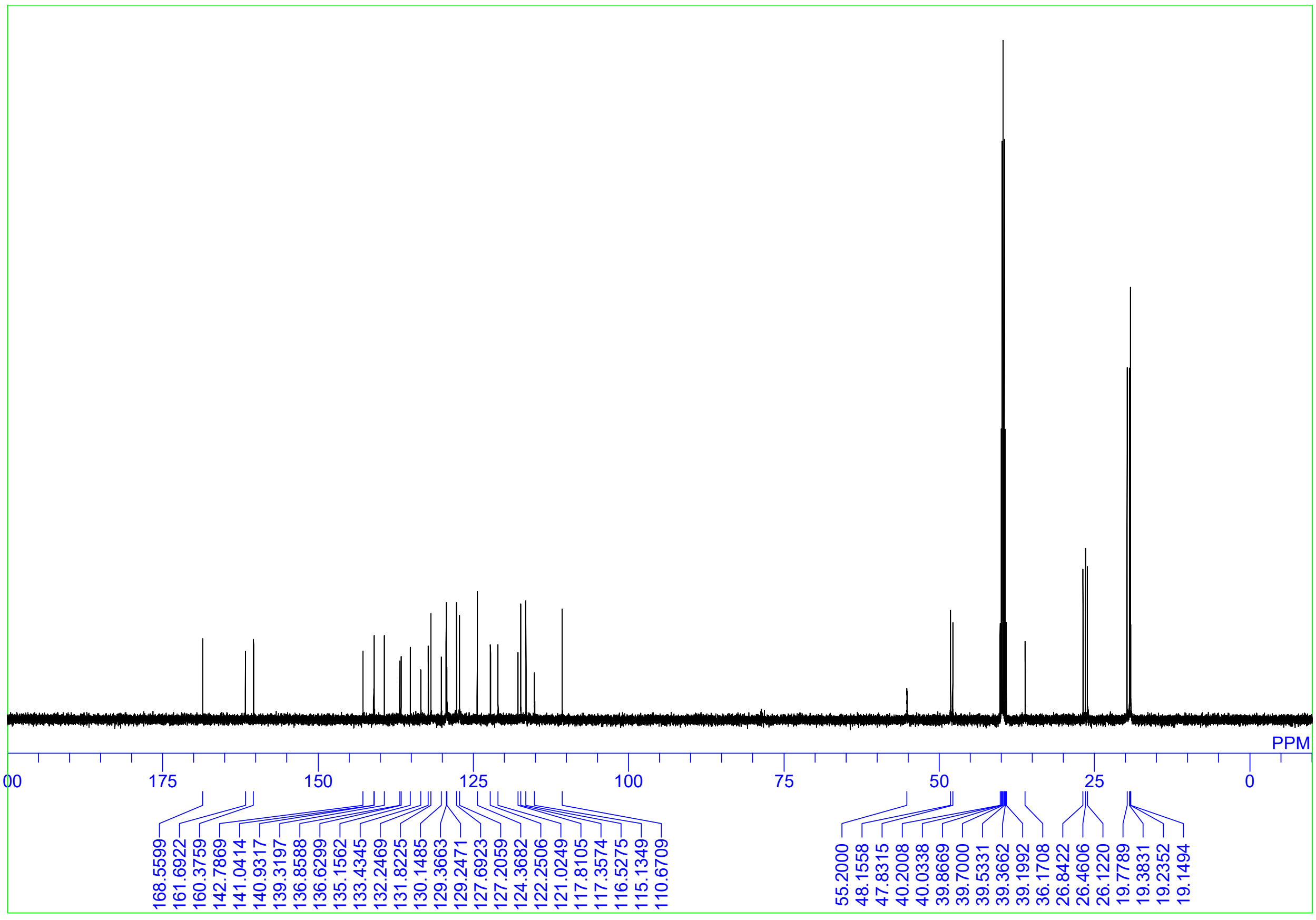

Supplement: Supplementary file 1 [file ao6c06995_si_006.pdf]
